# Patient Characteristics Associated With Occurrence of Preoperative Goals-of-Care Conversations

**DOI:** 10.1001/jamanetworkopen.2022.55407

**Published:** 2023-02-09

**Authors:** Kyung Mi Kim, Karleen F. Giannitrapani, Ariadna Garcia, Derek Boothroyd, Adela Wu, Raymond Van Cleve, Matthew D. McCaa, Maria Yefimova, Rebecca A. Aslakson, Arden M. Morris, Scott T. Shreve, Karl A. Lorenz

**Affiliations:** 1 Center for Innovation to Implementation (Ci2i), Veterans Affairs Palo Alto Health Care System, US Department of Veterans Affairs, Palo Alto, California; 2 Office of Research Patient Care Services, Stanford Health Care, Palo Alto, California; 3 Clinical Excellence Research Center, School of Medicine, Stanford University, Palo Alto, California; 4 Department of Social and Behavioral Sciences, School of Nursing, University of California San Francisco, San Francisco; 5 Primary Care and Population Health, School of Medicine, Stanford University, Palo Alto, California; 6 Quality Improvement Resource Center for Palliative Care, Stanford University, Palo Alto, California; 7 Quantitative Science Unit, School of Medicine, Stanford University, Palo Alto, California; 8 Department of Neurosurgery, Stanford Health Care, Palo Alto, California; 9 Center for Nursing Excellence and Innovation, UCSF Health, San Francisco, California; 10 Department of Physiological Nursing, School of Nursing, University of California San Francisco, San Francisco; 11 Department of Anesthesiology, Larner College of Medicine, University of Vermont, Burlington; 12 Veterans Affairs Palo Alto Health Care System, US Department of Veterans Affairs, Palo Alto, California; 13 S-SPIRE Center, Department of Surgery, School of Medicine, Stanford University, Palo Alto, California; 14 Department of Veterans Affairs, Washington, DC

## Abstract

**Importance:**

Communication about patients’ goals and planned and potential treatment is central to advance care planning. Undertaking or confirming advance care plans is also essential to preoperative preparation, particularly among patients who are frail or will undergo high-risk surgery.

**Objective:**

To evaluate the association between patient risk of hospitalization or death and goals-of-care conversations documented with a completed Life-Sustaining Treatment (LST) Decisions Initiative note among veterans undergoing surgery.

**Design, Setting, and Participants:**

This retrospective cross-sectional study included 190 040 veterans who underwent operations between January 1, 2017, and February 28, 2020. Statistical analysis took place from November 1, 2021, to November 17, 2022.

**Exposure:**

Patient risk of hospitalization or death, evaluated with a Care Assessment Need (CAN) score (range, 0-99, with a higher score representing a greater risk of hospitalization or death), dichotomized as less than 80 or 80 or more.

**Main Outcomes and Measures:**

Preoperative LST note completion (30 days before or on the day of surgery) or no LST note completion within the 30-day preoperative period prior to or on the day of the index operation.

**Results:**

Of 190 040 veterans (90.8% men; mean [SD] age, 65.2 [11.9] years), 3.8% completed an LST note before surgery, and 96.2% did not complete an LST note. In the groups with and without LST note completion before surgery, most were aged between 65 and 84 years (62.1% vs 56.7%), male (94.3% vs 90.7%), and White (82.2% vs 78.3%). Compared with patients who completed an LST note before surgery, patients who did not complete an LST note before surgery tended to be female (9.3% vs 5.7%), Black (19.2% vs 15.7%), married (50.2% vs 46.5%), and in better health (Charlson Comorbidity Index score of 0, 25.9% vs 15.2%); to have a lower risk of hospitalization or death (CAN score <80, 98.3% vs 96.9%); or to undergo neurosurgical (9.8% vs 6.2%) or urologic surgical procedures (5.9% vs 2.0%). Over the 3-year interval, unadjusted rates of LST note completion before surgery increased from 0.1% to 9.6%. Covariate-adjusted estimates of LST note completion indicated that veterans at a relatively elevated risk of hospitalization or death (CAN score ≥80) had higher odds of completing an LST note before surgery (odds ratio [OR], 1.29; 95% CI, 1.09-1.53) compared with those with CAN scores less than 80. High-risk surgery was not associated with increased LST note completion before surgery (OR, 0.93; 95% CI, 0.86-1.01). Veterans who underwent cardiothoracic surgery had the highest likelihood of LST note completion before surgery (OR, 1.35; 95% CI, 1.24-1.47).

**Conclusions and Relevance:**

Despite increasing LST note implementation, a minority of veterans completed an LST note preoperatively. Although doing so was more common among veterans with an elevated risk compared with those at lower risk, improving proactive communication and documentation of goals, particularly among higher-risk veterans, is needed. Doing so may promote goal-concordant surgical care and outcomes.

## Key Points

**Question:**

What is the association between patient risk of hospitalization or death, measured with a Care Assessment Need (CAN) score, and occurrence of goals-of-care conversations documented with a completed Life-Sustaining Treatment (LST) Decisions Initiative note among veterans who underwent surgery in the Veterans Health Administration?

**Findings:**

In this cross-sectional study, covariate-adjusted estimates of LST note completion indicated that veterans at high risk of hospitalization or death (CAN score ≥80) had a significantly higher likelihood of LST note completion before surgery.

**Meaning:**

This study suggests that a minority of veterans completed documentation of goals-of-care conversations preoperatively, despite a marginal increase in documentation of goals-of-care conversations associated with a higher risk of hospitalization or death among veterans who underwent operations.

## Teaser

This cross-sectional study evaluates the association between patient risk of hospitalization or death and goals-of-care conversations documented with a completed Life Sustaining Treatment Decisions Initiative note among individuals undergoing surgery in the Veterans Health Administration.

## Introduction

Surgical care is a critical component of health care^1^ and a substantial stressor.^2^ The need for surgery is extremely common among seriously ill older adults, and nearly one-third of older Americans face surgery even in the last year of life.^3^ Compared with their healthy counterparts, surgical patients who are elderly or hampered by multiple comorbid conditions face greater challenges during recovery and a higher risk of mortality and morbidity. Communication about goals, values, and future treatment options is crucial when patients are seriously ill or before important treatment decisions. Fostering communication about these issues is an important step to foster patient-centered, goal-concordant treatment.

Goals-of-care communication have the potential to promote better end-of-life care. Discussions about these topics are important to patients and surrogate decision makers who otherwise may feel anxiety about being in the surrogate role.^4^ Although a recent commentary notes that advance care planning has not yet demonstrated desired patient-family outcomes,^5^ clinical experience, observational studies, ethical principles, and expert opinion support the essential importance of proactive, high-quality communication about advance care planning.^4,5^ Undertaking and confirming goals and treatment plans, including those relevant to mortality, is especially needed in preoperative preparation, particularly among frail patients or those undergoing high-risk surgery.^6^

To foster better communication about important treatment decisions, the Veterans Health Administration (VHA) National Center for Ethics in Health Care instituted the Life-Sustaining Treatment (LST) Decisions Initiative (LSTDI) on July 1, 2018.^7^ The LSTDI is a national health care initiative that directly links conversations about goals of care (eg, values and goals) with clinician orders to implement and act on these values and goals. The LSTDI encompasses intervention elements including point-of-care triggering events for high-risk patients, clinical training, and a national electronic documentation template.^8,9^ The LSTDI can be completed in any Veterans Affairs VA care setting, and invasive procedures are designated as one of the triggering events prompting the LSTDI.^10^ We aimed to provide an overview of the use and timing of perioperative LST note completion and assess the association between patient risk of hospitalization or death and LST note completion among surgical patients.

## Methods

We conducted a retrospective cross-sectional analysis of all veterans who underwent a surgical procedure between January 1, 2017, and February 28, 2020, in the VHA. The study was approved by the joint VA Palo Alto Healthcare System and Stanford University institutional review boards. Patient consent was not required according to the joint authorization from VA Palo Alto Health Care System and Stanford University because the study comprised secondary analysis of archival data prior to the analysis. The study followed the Strengthening the Reporting of Observational Studies in Epidemiology (STROBE) reporting guideline for cross-sectional studies.

### Data and Study Cohort

We obtained data from the VA corporate data warehouse, and our analytic sample included 190 040 veterans. We defined surgical procedures as major diagnostic or therapeutic procedures using the Healthcare Cost and Utilization Project (HCUP) procedure class flags, sponsored by the Agency for Healthcare Research and Quality.^11^ The HCUP classifies *International Statistical Classification of Diseases and Related Health Problems, Tenth Revision, Procedure Coding System* (*ICD-10-PCS*) codes into 1 of 4 mutually exclusive categories, evaluated by clinical experts: minor diagnostic (nonoperating room procedures that are diagnostic), minor therapeutic (nonoperating room procedures that are therapeutic), major diagnostic (operating room procedures performed for diagnostic reasons), and major therapeutic (operating room procedures performed for therapeutic reasons).

**Figure 1.**
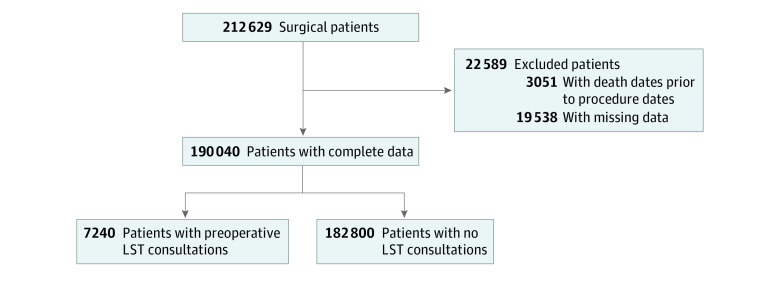
Flow Diagram of Sample Selection LST indicates Life-Sustaining Treatment.

We excluded patients who underwent nonoperating room procedures (ie, minor diagnostic and therapeutic procedures) from our study cohort. We also excluded those for whom the date of a procedure could not be determined and those with missing information on key study variables. Figure 1 shows the sample selection process.

### Outcome

The primary outcome was preoperative LST note completion. The policy requires goals-of-care conversations to include a minimum of 4 documented elements (ie, the patient’s decision-making capacity, goals of care, resuscitation preferences, and consent for the LST plan),^8^ which we define as LST note completion. These 4 required items from the LST note template are shown in eTable 1 in Supplement 1. Partial completion or incompletion of these data elements was defined as no LST note completion. Our primary analysis examined LST note completion within the preroperative window of 30 days prior to or on the day of the index operation. We created a binary timing measure indicating whether LST was completed within the preoperative window or not.

Starting 30 days before surgery aligns with the VA health care system’s policy for preoperative appointments.^12^ Reviewing goals of care or undertaking a new conversation within 1 month before surgery is appropriate for persons at high risk, even if their goals do not change. We examined the window of 30 days before surgery to reflect the LST note completion in the immediate perioperative context rather than the secular trend in general LST note completion.

### Independent Variable and Covariates

The VA health care system uses a Care Assessment Need (CAN) score, a predictive model that estimates prognosis and near-future health care use based on clinical and demographic data, which we used to identify veterans most likely to benefit from an LSTDI. We used CAN scores to define the threshold risk of hospitalization or death from up to 1 year prior to the index surgery. The CAN score is available for all patients who receive any primary care services in the VA health care system.^13^ CAN scores can identify veterans facing surgery who are at risk of higher mortality and morbidity.^13^ CAN scores range from 0 to 99, with a higher score representing a greater risk of hospitalization or death.^14^ A significant portion of patients with high CAN scores do not have a life-threatening illness and instead have a high CAN score as a result of serious mental illness or frequent admissions. Because CAN scores are updated weekly,^15^ we used the score most proximal to the surgery date.^7^ We confirmed a steep increase in hospitalization or death among patients with CAN scores of 80 or more, which we therefore defined as the high-risk threshold. CAN scores represent the associated risk as reported percentiles, not as an absolute risk. A CAN score of 80 represents a 13.6% probability of admission and a 4.1% probability of death in the next year.^14^

We controlled for known or hypothesized patient characteristics associated with completion of advance care planning or communication, including sex (female or male), race (Black, White, or other [Alaska Native, American Indian, Asian, Native Hawaiian, Pacific islander, or other]) and ethnicity (Latine or non-Latine) reported in the VA corporate database, age (18-54, 55-64, 65-84, and ≥85 years), marital status (married or divorced, widowed, separated or single, or never married), rurality (urban or rural), comorbidity score (Charlson Comorbidity Index [CCI] scores: 0 [best health], 1-3 [average health], or ≥4 [worst health]),^12^ health conditions (cancer, cardiopulmonary, dementia, end-stage kidney disease, end-stage liver disease, frailty, other conditions, or not available),^8,16,17^ surgical specialty (cardiothoracic surgery, general surgery, neurosurgery, orthopedic surgery, urologic surgery, vascular surgery, or other), risk of surgery (high-risk procedure vs non–high-risk procedure),^18^ case type (elective vs nonelective), and fixed effects for procedure year and hospital. We used clinical classifications software developed by the HCUP^19^ to categorize operations into specialty groups. Two of us (K.M.K. and A.W.) with clinical expertise in surgery reviewed these categories to ensure they aligned with the actual clinical practice. Disagreements were reviewed by a third coauthor (R.A.A.) and were resolved by consensus, and we created a 7-category surgical specialty variable as indicated above. Otorhinolaryngology, gynecology, transplant, and endocrine procedures were combined into a single convenience category (other) because of the small sample sizes.

We identified surgical risk using the list of high-risk operations developed by previous researchers.^18^ Originally, high-risk operations were identified using *International Classification of Diseases, Ninth Revision, Clinical Modification* (*ICD-9-CM*) codes. Because *ICD* codes were transited from *ICD-9-CM* to *ICD-10-PCS* codes in the fourth quarter of 2015, we converted *ICD-9-CM* codes to *ICD-10-PCS* codes aligned with our study period (2017-2020) using the equivalence mapping developed by the Centers for Medicare & Medicaid Services^20^ and the conversion files developed by the National Bureau of Economic Research.^21^ The full list of converted *ICD-10-PCS* codes are available in eTable 2 in Supplement 1.

### Statistical Analysis

Statistical analysis took place from November 1, 2021, to November 17, 2022. We used descriptive statistics to summarize patient characteristics by LST note completion status. We used standardized mean differences to compare 2 groups because they are less sensitive to large sample sizes than tests of significance.^22^ Next, we examined the unadjusted LST note completion patterns by calculating the proportion of surgical patients who completed LST notes quarterly, from January 2017 to February 2020, prior to the COVID-19 pandemic.

We assessed the potential collinearity of the variables using Pearson correlation coefficients (ie, CAN score and health conditions, CAN score and CCI score, and health conditions and CCI score) before we accounted for all 3 variables in the model. Then, we examined the association between CAN scores and the timing of LST note completion using multivariable logistic regression, adjusting for all covariates listed above. We estimated adjusted odds ratios (ORs) with 95% CIs and calculated the estimated probability of LST note completion using marginal standardization.^23^ To compare the variations in LST note completion among the subgroups of veterans more likely to have LST note completion, we estimated the covariate-adjusted standardized LST note completion rates by calculating the ratio of observed to expected LST note completion and multiplying that number by the overall LST note completion rate.^24,25^

We conducted sensitivity analyses to assess the robustness of our results. This included repeating the main analyses using an extended cohort with a completed LST note within 90 days before surgery, excluding patients who had LST note completion outside of 30 days before the surgery window, and excluding VA facilities with no LST note completion.

Analyses were performed using R, version 4.0.5 (R Group for Statistical Computing). Probability and covariate-adjusted standardized rates^26^ were estimated using Stata MP, version 17.0 (StataCorp LLC). All *P* values were from 2-sided tests, and results were deemed statistically significant at *P* < .05.

## Results

Of 190 040 veterans (90.8% men and 9.2% women; mean [SD] age, 65.2 [11.9] years), 3.8% completed an LST note before surgery, and 96.2% did not complete an LST note. In the groups with and without LST note completion before surgery, most were aged between 65 and 84 years (62.1% vs 56.7%), male (94.3% vs 90.7%), and White (82.2% vs 78.3%) (Table 1). Compared with patients who completed an LST note before surgery, patients who did not complete an LST note before surgery tended to be female (9.3% vs 5.7%), Black (19.2% vs 15.7%), married (50.2% vs 46.5%), and in better health (CCI score of 0, 25.9% vs 15.2%); to have a lower risk of hospitalization or death (CAN score <80, 98.3% vs 96.9%); or to undergo neurosurgical (9.8% vs 6.2%) or urologic surgical procedures (5.9% vs 2.0%).

**Table 1.** Characteristics of Surgical Patients in the Veterans Affairs Medical Center, January 2017 to February 2020

Characteristic	Patients, No. (%)		Standardized mean difference^a^
	No preoperative LST note completion (n = 182 800)	Preoperative LST note completion (n = 7240)	
Age group, y			
18-54	28 086 (15.4)	655 (9.0)	0.31
55-64	44 893 (24.6)	1451 (20.0)	
65-84	103 689 (56.7)	4495 (62.1)	
≥85	6132 (3.4)	639 (8.8)	
Sex			
Female	17 060 (9.3)	416 (5.7)	0.14
Male	165 740 (90.7)	6824 (94.3)	
Race			
Black	35 047 (19.2)	1138 (15.7)	0.10
White	143 115 (78.3)	5948 (82.2)	
Other^b^	4638 (2.5)	154 (2.1)	
Ethnicity			
Latine	9696 (5.3)	309 (4.3)	0.05
Non-Latine	173 104 (94.7)	6931 (95.7)	
Marital status			
Married	91 736 (50.2)	3369 (46.5)	0.08
Divorced, widowed, or separated	70 216 (38.4)	3053 (42.2)	
Single or never married	20 848 (11.4)	818 (11.3)	
CAN score			
<80	179 717 (98.3)	7018 (96.9)	0.09
≥80	3083 (1.7)	222 (3.1)	
CCI score			
0 (Best health)	47 362 (25.9)	1099 (15.2)	0.40
1-3 (Average health)	81 211 (44.4)	2690 (37.2)	
≥4 (Worst health)	54 227 (29.7)	3451 (47.7)	
Health conditions^c^			
ESKD	6406 (3.5)	445 (6.1)	0.35
Cardiopulmonary disease	35 980 (19.7)	1923 (26.6)	
Cancer	45 995 (25.2)	1974 (27.3)	
Dementia	399 (0.2)	42 (0.6)	
Frailty^d^	23 002 (12.6)	1083 (15.0)	
Other	31 339 (17.1)	915 (12.6)	
Not available	39 679 (21.7)	858 (11.9)	
Risk of surgery			
Non–high risk	137 566 (75.3)	5599 (77.3)	0.05
High risk	45 234 (24.7)	1641 (22.7)	
Surgical specialty			
General surgery	44 837 (24.5)	1876 (25.9)	0.29
Cardiothoracic surgery	28 728 (15.7)	1512 (20.9)	
Neurosurgery	17 948 (9.8)	446 (6.2)	
Orthopedic surgery	60 887 (33.3)	2643 (36.5)	
Urologic surgery	10 704 (5.9)	146 (2.0)	
Vascular surgery	11 821 (6.5)	441 (6.1)	
Other	7875 (4.3)	176 (2.4)	
Case type^e^			
Elective	114 332 (62.5)	5982 (82.6)	0.46
Emergency	68 468 (37.5)	1258 (17.4)	
Rurality			
Urban	115 150 (64.0)	4776 (66.0)	0.06
Rural	61 868 (34.4)	2326 (32.1)	
Highly rural	2821 (1.6)	135 (1.9)	
Continental US	85 (0.05)	1 (0.01)	
Not available	26 (0.01)	2 (0.03)	

Abbreviations: CAN, Care Assessment Need; CCI, Charlson Comorbidity Index; ESKD, end-stage kidney disease; LST, Life-Sustaining Treatment.

^a^ The reported standardized mean difference for each variable is the maximum of both pairwise standardized mean differences. Standardized mean differences between 0.2 and less than 0.5, 0.5 and 0.8, and greater than 0.8 are considered small, medium, and large, respectively.

^b^ Includes veterans identifying as Alaska Native, American Indian, Asian, Native Hawaiian, Pacific Islander, or other.

^c^ We used a set of mutually exclusive, hierarchical clinical diagnoses of health conditions.

^d^ Conditions to define frailty included at least 1 of the following conditions: stroke, Alzheimer disease, dementia, acute delirium, Parkinson disease, hip fracture, incontinence, pneumonia, dehydration, syncope, or leg cellulitis.

^e^ Cases that started between 7 pm and 7 am were defined as emergencies.

**Figure 2.**
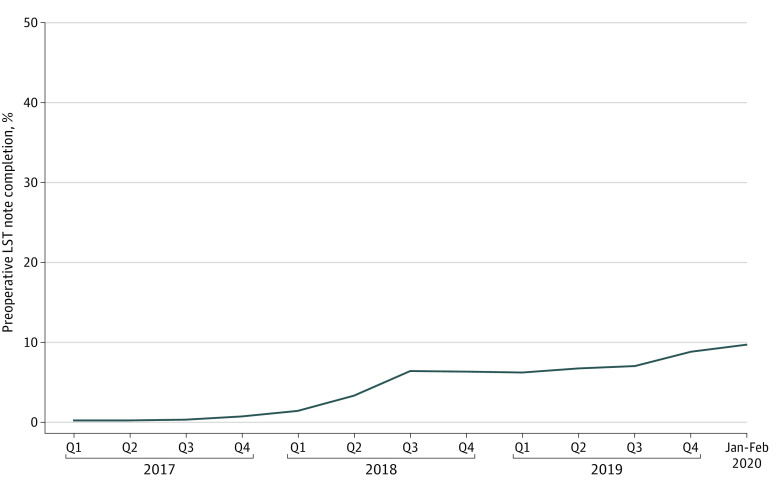
Unadjusted Proportion of Surgical Patients Who Completed a Life-Sustaining Treatment (LST) Note Quarterly (Q1-Q4), January 2017 to February 2020

Unadjusted rates of LST note completion increased over time, from 0.1% during the first quarter of 2017 to 9.6% during January and February of 2020 (Figure 2). For those who completed an LST note before surgery, the mean (SD) completion time was 5.2 (7.4) days before surgery.

Covariate-adjusted estimates for LST note completion indicate that patients who were at high risk of hospitalization or death (ie, CAN score ≥80) had 1.3 times higher odds of LST note completion before surgery (OR, 1.29; 95% CI, 1.09-1.53) compared with those with CAN scores less than 80 (Table 2). Patient age of 85 years or older was associated with an increase in LST note completion before surgery (OR, 2.84; 95% CI, 2.47-3.25). High-risk surgery was not associated with increased LST note completion before surgery (OR, 0.93; 95% CI, 0.86-1.01). Patients with the worst health status (ie, CCI score ≥4) were more likely to have LST note completion before surgery (OR, 2.54; 95% CI, 2.18-2.97). Patients who underwent cardiothoracic surgery were more likely to complete an LST note before surgery than those who underwent other types of operations (OR, 1.35; 95% CI, 1.24-1.47) (Table 2). Patients who underwent urologic surgery had the least likelihood of LST note completion before surgery relative to those who underwent general surgery (OR, 0.23; 95% CI, 0.19-0.28).

**Table 2.** Adjusted Analyses of Association of Patient Characteristics With the Timing of LST Note Completion^a^

Characteristic	Veterans with preoperative LST note completion	
	Odds ratio (95% CI)	Absolute difference, % (95% CI)
Age group, y		
18-54	1 [Reference]	1 [Reference]
55-64	1.04 (0.94 to 1.16)	0.12 (−0.18 to 0.42)
65-84	1.13 (1.03 to 1.25)	0.36 (0.08 to 0.64)
≥85	2.84 (2.47 to 3.25)	4.13 (3.54 to 4.72)
Sex		
Female	1 [Reference]	1 [Reference]
Male	1.13 (1.01 to 1.27)	0.37 (0.04 to 0.70)
Race		
Black	0.89 (0.82 to 0.96)	−0.36 (−0.59 to −0.13)
White	1 [Reference]	1 [Reference]
Other^b^	1.04 (0.87 to 1.25)	0.14 (−0.43 to 0.70)
Ethnicity		
Non-Latine	1 [Reference]	1 [Reference]
Latine	1.01 (0.88 to 1.15)	0.02 (−0.40 to 0.44)
Marital status		
Married	1 [Reference]	1 [Reference]
Divorced, widowed, or separated	1.19 (1.13 to 1.26)	0.54 (0.37 to 0.71)
Single or never married	1.24 (1.13 to 1.36)	0.66 (0.37 to 0.95)
CAN score		
<80	1 [Reference]	1 [Reference]
≥80	1.29 (1.09 to 1.53)	0.85 (0.25 to 1.44)
CCI score		
0 (Best health)	1 [Reference]	1 [Reference]
1-3 (Average health)	1.27 (1.09 to 1.48)	0.58 (0.22 to 0.94)
≥4 (Worst health)	2.54 (2.18 to 2.97)	2.92 (2.50 to 3.35)
Health conditions^c^		
Cancer	1 [Reference]	1 [Reference]
ESKD	1.67 (1.47 to 1.90)	1.81 (1.30 to 2.32)
Cardiopulmonary disease	1.28 (1.19 to 1.38)	0.80 (0.55 to 1.04)
Dementia	2.59 (1.76 to 3.81)	3.88 (1.82 to 5.93)
Frailty^d^	1.38 (1.26 to 1.52)	1.07 (0.76 to 1.38)
Other	0.77 (0.70 to 0.85)	−0.70 (−0.95 to −0.45)
Not available	0.77 (0.65 to 0.91)	−0.71 (−1.14 to −0.27)
Risk of surgery		
Non–high risk	1 [Reference]	1 [Reference]
High risk	0.93 (0.86 to 1.01)	−0.21 (−0.43 to 0.02)
Surgical specialty		
General surgery	1 [Reference]	1 [Reference]
Cardiothoracic surgery	1.35 (1.24 to 1.47)	1.08 (0.76 to 1.39)
Neurosurgery	0.76 (0.68 to 0.86)	−0.82 (−1.16 to −0.49)
Orthopedic surgery	0.94 (0.87 to 1.01)	−0.21 (−0.44 to 0.03)
Urologic surgery	0.23 (0.19 to 0.28)	−3.00 (−3.26 to −2.74)
Vascular surgery	0.57 (0.50 to 0.64)	−1.55 (−1.86 to −1.25)
Other	0.57 (0.48 to 0.69)	−1.53 (−1.96 to −1.10)
Case type^e^		
Emergency	1 [Reference]	1 [Reference]
Elective	3.23 (2.99 to 3.49)	3.11 (2.93 to 3.29)
Rurality		
Urban	1 [Reference]	1 [Reference]
Rural	0.83 (0.78 to 0.89)	−0.55 (−0.73 to −0.36)
High rural	0.86 (0.70 to 1.05)	−0.45 (−1.03 to 0.13)
Continental US	1.22 (0.15 to 9.80)	0.69 (−6.84 to 8.22)
Not available	1.12 (0.23 to 5.35)	0.36 (−4.99 to 5.71)
Year		
2017	1 [Reference]	1 [Reference]
2018	25.26 (21.45 to 29.76)	4.31 (4.16 to 4.47)
2019	45.88 (38.97 to 54.02)	6.99 (6.79 to 7.20)
2020	57.13 (46.88 to 69.62)	8.27 (7.54 to 9.00)

Abbreviations: CAN, Care Assessment Need; CCI, Charlson Comorbidity Index; ESKD, end-stage kidney disease; LST, Life-Sustaining Treatment.

^a^ The estimated probabilities were calculated from the adjusted model using the Margins command in Stata. All other analyses were performed in R.

^b^ Includes veterans identifying as Alaska Native, American Indian, Asian, Native Hawaiian, Pacific Islander, or other.

^c^ We used a set of mutually exclusive, hierarchical clinical diagnoses of health conditions.

^d^ Conditions to define frailty included at least 1 of the following conditions: stroke, Alzheimer disease, dementia, acute delirium, Parkinson disease, hip fracture, incontinence, pneumonia, dehydration, syncope, or leg cellulitis.

^e^ Cases that started between 7 pm and 7 am were defined as emergencies.

**Figure 3.**
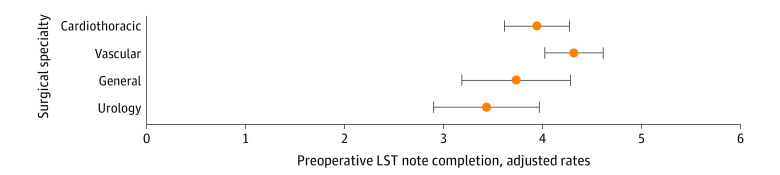
Adjusted Rates of Preoperative Life-Sustaining Treatment (LST) Note Completion by Surgical Specialty Among Veterans 65 Years or Older With Cancer Diagnosis Who Underwent Elective High-risk Procedure The error bars indicate 95% CIs.

Covariate-adjusted standardized rates confirm that the chance of LST note completion was greater among veterans of older age who underwent elective surgery with more comorbidities but also indicate variations by surgical specialty (Figure 3). Rates of LST note completion in patients with a cancer diagnosis were about 90% lower among those who underwent high-risk elective urologic surgery (adjusted standardized rate, 3.44; 95% CI, 2.90-3.96) than those who underwent high-risk elective vascular surgery (adjusted standardized rate, 4.32; 95% CI, 4.03-4.61).

Sensitivity analyses showed consistent results. Using the 91- vs 31-day interval to estimate LST note completion preoperatively increased our unadjusted estimate of preoperative LST note completion from 3.8% to 4.7%. Covariate-adjusted estimates excluding those who had already completed LST note documentation within a year before the index operation or excluding hospitals with no preoperative LST note completion were also consistent, indicating that increased LST note completion was associated with higher risk of hospitalization or death (ie, CAN score ≥80), higher CCI score, existing health conditions, and older age.

## Discussion

We evaluated whether risk of hospitalization or death and other patient characteristics were associated with the timing of goals-of-care completion measured with the VA health care system’s LST note documentation. We found that, consistent with a prior study that found a low use of perioperative palliative care,^12^ most surgical patients did not have a completed LST note. Although the number of patients completing a preoperative LST note increased from 2017 to early 2020, perioperative LST note completion rates overall were very low among veterans undergoing surgery. Among those who had a completed LST note, LST notes were documented more often before surgery among veterans with a high risk of hospitalization or death.

Our findings revealed specific opportunities to improve LST note completion. Although our result suggests that patients with an elevated risk of hospitalization or death (CAN score ≥80) had a marginally increased likelihood of LST note completion before surgery, veterans who underwent high-risk surgical procedures were not more likely to complete an LST note before surgery. Our investigation of variations in LST note completion by surgical specialty might shed light on the potential explanation for this counterintuitive association between risk of surgery and preoperative LST note completion. First, perceptions of high-risk procedures might need to be refocused in the VA health care system. The current focus of high-risk procedures is more centered on cardiothoracic, vascular, and general surgical procedures, as confirmed in our subgroup analysis. However, the urology service performed more high-risk procedures than did any other service (80% of urology procedures were classified as high-risk and 84% of urology patients had a cancer diagnosis) in the VA health care system, yet the preoperative LST note documentation was the lowest among urology patients. In addition, one-third of urology patients were classified as having the poorest level of health (CCI score ≥4), similar to cardiothoracic, vascular, and general surgery patients. Clinician education and training for surgical specialties other than cardiothoracic and vascular surgery might be needed to improve LST note discussions. Second, the patients’ characteristics in each service might be associated with surgeons’ varying perception of the necessity of goals-of-care conversations. For instance, orthopedic surgeons performed the lowest proportion of high-risk procedures but completed the second-highest proportion of preoperative LST notes. The factors improving this service’s documentation could be that orthopedics serves the most elderly patients (4.6% of whom are aged ≥85 years, whereas 1.4% of urology patients are aged ≥85 years) and that clinicians in orthopedic services might identify their patients’ needs more consistently.

Given that the LST note completion rates in the perioperative setting were lower than those of veterans in general,^8^ LST notes might not be designed most appropriately to serve surgical needs. Surgical teams might therefore use generic notes rather than LST notes to document discussions about goals of care, as they do with discussions of the risks and benefits of surgery. However, if that is the case, embedding patient preferences and goals of care in a generic note rather than in an LST note could hinder access to this information for other clinicians. In addition, use of a generic note diminishes the advantageous feature of an LSTDI, which enables clinicians to order an intervention based on patients’ goals of care. A modification of LST notes to reflect specific needs of surgical preparation might improve surgical goals-of-care documentation.

In addition to patient-level characteristics, a complex interplay of practice and organizational factors may be associated with the limited implementation of goals-of-care communication in perioperative care. In general, this includes the high value that surgical practice places on mortality avoidance, and in settings other than the VA health system, the surgical payment model may increase surgical overtreatment.^27^ Barriers to goals-of-care conversations in nonsurgical settings that might also be salient to the surgical context include limited time and resources, lack of clinician readiness, an unfavorable organizational climate, and low leadership involvement in promoting change.^28^

Future studies examining outcomes associated with the occurrence and timing of perioperative goals-of-care communication that evaluate patients’ experiences and values, such as patient-reported outcomes and quality of communication,^12^ are needed in the perioperative setting. Potential benefits of effective preoperative communication might include the use of modified surgical approaches or better postoperative surveillance for higher-risk patients. Prognostic awareness might ease the emotional burden of undesirable outcomes on caregivers.

### Limitations

This study was subject to several limitations. The CAN score, our measure of mortality risk, has been validated as informative in the surgical context but may not be as broadly and specifically informative as other estimation tools, such as the Risk Analysis Index.^29^ In addition, we examined a limited perioperative window; however, sensitivity analyses broadening preoperative consideration to 90 days did not change our results, and we focused on a narrow rather than broad time window to reflect the surgical team’s performance. Because we could not identify specific clinicians completing LST notes, we conservatively attributed completion to the surgical team, although this may overestimate their involvement. Surgical teams might document goals-of-care conversations in documents other than an LST note, which may underestimate the discussions performed by surgical teams. The surgeon plays a critical role in the goals-of-care discussion, and it is a limitation of this study that we were not able to account for clinician variation, although we controlled for the secular trends in hospitals. Our sample was limited to veterans, and the VA health system has extensive resources and support for palliative care; therefore, these findings may also overestimate the use of perioperative goals-of-care communication in non–VA health system settings.

## Conclusions

In this cross-sectional study of veterans, LST note completion before surgery was more common among veterans with an elevated risk compared with those at lower risk of hospitalization or mortality, a complex risk that the CAN score represents. However, a small minority of veterans undergoing surgery completed LST notes perioperatively despite a policy designed to increase LST note implementation. The study results highlight variations in LST note completion across surgical specialties, and surgical services performing high-risk procedures do not align with the high LST note completion group but emphasize the need to improve documentation of patient-caregiver goals in the perioperative setting. Promoting proactive perioperative communication to reflect potential changes in the patient’s preferences in treatment choices, particularly among veterans at higher risk, is also warranted. Additional studies are needed to characterize the potential of such communication to improve outcomes such as early palliative care involvement when morbidity occurs, improved transitional planning, and the preparation and experience of caregivers.

## Notes

Supplement 1. **eTable 1.** LST Template Questions Used to Define a Conversation About Goals For Care **eTable 2.** *ICD 9-CM* and *ICD-10-PCS* Codes to Identify High-Risk Surgery

Supplement 2.
Data Sharing Statement
