# Supplementary material for: Patient Characteristics Associated With Occurrence of Preoperative Goals-of-Care Conversations
Source: JAMA Netw Open. 2023 Feb 9;6(2):e2255407. doi: 10.1001/jamanetworkopen.2022.55407 (PMC9912129; doi:10.1001/jamanetworkopen.2022.55407)
Supplement: Supplement 1. — eTable 1. LST Template Questions Used to Define a Conversation About Goals For Care eTable 2. ICD 9-CM and ICD-10-PCS Codes to Identify High-Risk Surgery [file jamanetwopen-e2255407-s001.pdf]

Supplementary Online Content

Kim KM, Giannitrapani KF, Garcia A, et al. Patient characteristics associated with occurrence of preoperative goals-of-care conversations. *JAMA Netw Open.* 2023;6(2):e2255407. doi:10.1001/jamanetworkopen.2022.55407

eTable 1. LST Template Questions Used to Define a Conversation About Goals For Care
eTable 2. *ICD 9-CM* and *ICD-10-PCS* Codes to Identify High-Risk Surgery

This supplementary material has been provided by the authors to give readers additional information about their work.

© 2023 Kim KM et al. *JAMA Network Open.*

eTable 1. LST Template Questions Used to Define a Conversation About Goals For Care[a]

LIFE-SUSTAINING TREATMENT	
**1. Does the patient have capacity to make decisions about life-sustaining treatment?**	☐ The patient has capacity to make decisions about life-sustaining treatments.<br>☐ The patient lacks capacity to make decisions about life-sustaining treatments and has a surrogate.<br>☐ The patient lacks capacity to make decisions about life-sustaining treatments and has no surrogate.
2. Who is the person authorized under VA policy to make decisions for the patient if/when the patient loses decision-making capacity?	☐ Authorized surrogate if/when the patient loses decision-making capacity:<br>☐ The patient has no surrogate authorized to make health care decisions if/when the patient loses decision-making capacity.
3. Have you reviewed available documents that reflect the patient's wishes regarding life-sustaining treatments? Example: advance directives, state-authorized portable orders (e.g., POLST, MOST), Life-Sustaining Treatment notes/orders.	☐ No advance directive, state-authorized portable orders (e.g., POLST, MOST), or Life-Sustaining Treatment notes/orders were available in the record or presented by the patient (or surrogate).<br>☐ I reviewed with the patient (or surrogate) all active advance directive(s), state-authorized portable orders (e.g., POLST, MOST), or Life-Sustaining Treatment notes/orders available in the record or presented by the patient (or surrogate).
4. Does the patient (or surrogate) have sufficient understanding of the patient's medical condition to make informed decisions about life-sustaining treatments?	☐ Yes. The patient's (or surrogate's) understanding is consistent with the medical facts.<br>☐ Other (e.g., the patient lacks decision-making capacity and has no surrogate)
**5. What are the patient's goals of care? (Select all that apply. Do not attempt to rank the goals of care here)**	Patient's goals of care in their own words, or as stated by the surrogate:<br>☐ To be cured of:<br>☐ To prolong life<br>☐ To improve or maintain function, independence, quality of life<br>☐ To be comfortable<br>☐ To obtain support for family/caregiver<br>☐ To achieve life goals, including:
6. What is the current plan for use of life-sustaining treatment?	☐ Full scope of treatment in circumstances other than cardiopulmonary arrest.<br>☐ Limit life-sustaining treatment, as follows:<br>*Artificial Nutrition*<br>o No artificial nutrition (enteral or parenteral)<br>o Limit artificial nutrition as follows:<br>*Artificial Hydration*

© 2023 Kim KM et al. *JAMA Network Open.*

	○ No artificial hydration (enteral, IC, or subcutaneous)<br>○ Limit artificial hydration as follows:<br>*Mechanical Ventilation*<br>○ No invasive mechanical ventilation (e.g., CPAP, BiPAP)<br>○ Limit mechanical ventilation as follows:<br>*Transfers between Levels of Care*<br>○ No transfers to the ICU except if need for comfort<br>○ No transfers to the hospital except if needed for comfort<br>○ Limit transfers as follows (e.g., patient wishes to remain at home if possible):<br>*Limit Other Life-Sustaining Treatment as follows (e.g., blood products, dialysis):*<br>☐ No Life-Sustaining Treatment in circumstances other than cardiopulmonary arrest.<br><br>**Cardiopulmonary resuscitation (CPR)**<br>☐ **Full Code: Attempt CPR**<br>☐ **DNAR/DNR: Do not attempt CPR**<br>☐ **DNAR/DNR with exception: ONLY attempt CPR during the following procedure:**
7. Who participated in this discussion?	Document participants and other relevant information:
**8. Who has given oral informed consent for the life-sustaining treatment plan outlined above?**	☐ The patient has given oral informed consent for the life-sustaining treatment plan.<br>☐ The surrogate has given oral informed consent for the life-sustaining treatment plan. Name of the surrogate providing consent:<br>☐ The patient lacks decision-making capacity and has no surrogate.<br>○ The LST plan has been approved through the multidisciplinary committee review process.

[a]Items in bold are minimum documented elements of LST template.

© 2023 Kim KM et al. *JAMA Network Open.*

eTable 2. *ICD 9-CM* and *ICD-10-PCS* Codes to Identify High-Risk Surgery[b]

Speciality	Modified CCS description	Name of procedure	ICD9 CM	ICD10 PCS
Neuro	Incision and excision of CNS	other craniotomy	1.24	00J00ZZ
				00W00JZ
				00W00KZ
				0N800ZZ
				0N803ZZ
				0N804ZZ
				0NC10ZZ
				0NC13ZZ
				0NC14ZZ
				0NC20ZZ
				0NC23ZZ
				0NC24ZZ
				0NC30ZZ
				0NC33ZZ
				0NC34ZZ
				0NC40ZZ
				0NC43ZZ
				0NC44ZZ
				0NC50ZZ
				0NC53ZZ
				0NC54ZZ
				0NC60ZZ
				0NC63ZZ
				0NC64ZZ
				0NC70ZZ
				0NC73ZZ
				0NC74ZZ
				0NC80ZZ
				0NC83ZZ
				0NC84ZZ
				0NH00MZ
				0NH03MZ
				0NH04MZ
				0NP000Z
				0NP004Z
				0NP005Z
				0NP007Z
				0NP00KZ
				0NP00SZ
				0NP030Z
				0NP034Z
				0NP037Z
				0NP03KZ
				0NP03SZ
				0NP040Z
				0NP044Z
				0NP047Z
				0NP04KZ
				0NP04SZ
				0NP0X4Z

© 2023 Kim KM et al. *JAMA Network Open.*

		0NP0XSZ<br>0NW000Z<br>0NW004Z<br>0NW005Z<br>0NW007Z<br>0NW00JZ<br>0NW00KZ<br>0NW00MZ<br>0NW00SZ<br>0NW030Z<br>0NW034Z<br>0NW035Z<br>0NW037Z<br>0NW03JZ<br>0NW03KZ<br>0NW03MZ<br>0NW03SZ<br>0NW040Z<br>0NW044Z<br>0NW045Z<br>0NW047Z<br>0NW04JZ<br>0NW04KZ<br>0NW04MZ<br>0NW04SZ<br>0W9100Z<br>0W910ZZ<br>0WC10ZZ<br>0WC13ZZ<br>0WC14ZZ<br>0WH10YZ<br>0WH13YZ<br>0WH14YZ<br>0WJ10ZZ<br>0WP100Z<br>0WP101Z<br>0WP10JZ<br>0WP10YZ<br>0WP130Z<br>0WP131Z<br>0WP13JZ<br>0WP13YZ<br>0WP140Z<br>0WP141Z<br>0WP14JZ<br>0WP14YZ<br>0WW100Z<br>0WW101Z<br>0WW103Z<br>0WW10JZ<br>0WW10YZ<br>0WW130Z<br>0WW131Z<br>0WW133Z

				0WW13JZ
				0WW13YZ
				0WW140Z
				0WW141Z
				0WW143Z
				0WW14JZ
				0WW14YZ
		other craniectomy	1.25	0N500ZZ
				0N503ZZ
				0N504ZZ
				0NB00ZZ
				0NB03ZZ
				0NB04ZZ
				0NT10ZZ
				0NT20ZZ
				0NT30ZZ
				0NT40ZZ
				0NT50ZZ
				0NT60ZZ
				0NT70ZZ
		incise cerebral meninges	1.31	009100Z
				00910ZZ
				00C10ZZ
				00C13ZZ
				00C14ZZ
		other brain incision	1.39	009000Z
				00900ZZ
				009030Z
				00903ZZ
				009040Z
				00904ZZ
				00C00ZZ
				00C03ZZ
				00C04ZZ
				00H002Z
				00H003Z
				00H032Z
				00H033Z
				00H042Z
				00H043Z
				00H602Z
				00H603Z
				00H632Z
				00H633Z
				00H642Z
				00H643Z
				00P000Z
				00P002Z
				00P003Z
				00P007Z
				00P00JZ
				00P00KZ
				00P030Z
				00P032Z

© 2023 Kim KM et al. *JAMA Network Open.*

				00P033Z<br>00P037Z<br>00P03JZ<br>00P03KZ<br>00P040Z<br>00P042Z<br>00P043Z<br>00P047Z<br>00P04JZ<br>00P04KZ<br>00P600Z<br>00P602Z<br>00P603Z<br>00P630Z<br>00P632Z<br>00P633Z<br>00P640Z<br>00P642Z<br>00P643Z<br>00P6X2Z<br>00W000Z<br>00W002Z<br>00W003Z<br>00W007Z<br>00W00MZ<br>00W030Z<br>00W032Z<br>00W033Z<br>00W037Z<br>00W03JZ<br>00W03KZ<br>00W03MZ<br>00W040Z<br>00W042Z<br>00W043Z<br>00W047Z<br>00W04JZ<br>00W04KZ<br>00W04MZ<br>00W600Z<br>00W602Z<br>00W603Z<br>00W60MZ<br>00W630Z<br>00W632Z<br>00W633Z<br>00W63MZ<br>00W640Z<br>00W642Z<br>00W643Z<br>00W64MZ
		ex cereb meningeal les	1.51	00510ZZ<br>00513ZZ<br>00514ZZ

© 2023 Kim KM et al. *JAMA Network Open.*

				00B10ZZ
				00B13ZZ
				00B14ZZ
				00D10ZZ
				00D13ZZ
				00D14ZZ
		brain lobectomy	1.53	08QM3ZZ
				08QL0ZZ
				08QL3ZZ
				08QM0ZZ
		other brain excision	1.59	08QL3ZZ
				08QM3ZZ
				08QL0ZZ
				08QM0ZZ
Otolaryngologic	Pineal & pituitary	exc pituit les-transfron	7.61	0G800ZZ
				0G803ZZ
				0G804ZZ
				0GB00ZZ
Thoracic	Lobectomy or pneumonectomy	lobectomy of lung#	32.40	
		complete pneumonectomy#	32.50	0BTL4ZZ
				0BTK4ZZ
				0BTM4ZZ
		emphysema bleb plication	32.21	0BQK4ZZ
				0BQM7ZZ
				0BQK8ZZ
				0BQL7ZZ
				0BQM3ZZ
				0BQK7ZZ
				0BQM8ZZ
				0BQM0ZZ
				0BQL0ZZ
				0BQL3ZZ
				0BQL8ZZ
				0BQL4ZZ
				0BQK0ZZ
				0BQK3ZZ
				0BQM4ZZ
		lung vol reduction surg	32.22	0BBK3ZZ
				0B5K7ZZ
				0BBM7ZZ
				0B5K3ZZ
				0BBL0ZZ
				0B5M0ZZ
				0B5L0ZZ
				0BBL3ZZ
				0B5M3ZZ
				0BBK0ZZ
				0B5M7ZZ
				0BBM3ZZ
				0BBL7ZZ
				0B5L7ZZ
				0B5K0ZZ

© 2023 Kim KM et al. *JAMA Network Open.*

		partial lobectomy of lung	32.30	0B5L3ZZ<br>0BBM0ZZ<br>0BBK7ZZ<br>0BBG4ZZ<br>0BBD4ZZ<br>0BBC4ZZ<br>0BBL4ZZ<br>0BTH4ZZ<br>0BBF4ZZ<br>0BBK4ZZ<br>0BBJ4ZZ<br>0BBH4ZZ
Otolaryngologic	Major Larynx procedures	complete laryngectomy	30.3	0B110F4<br>0B110Z4<br>0B113F4<br>0B113Z4<br>0B114F4<br>0B114Z4<br>0CTS0ZZ<br>0CTS0ZZ<br>0CTS4ZZ<br>0CTS4ZZ<br>0CTS7ZZ<br>0CTS7ZZ<br>0CTS8ZZ<br>0CTS8ZZ
Otolaryngologic	Major Tracheal/bronchial	bronchial repair nec	33.48	0BV63ZZ<br>0BU84JZ<br>0BV54ZZ<br>0BV53CZ<br>0BV98DZ<br>0BV64DZ<br>0BV40ZZ<br>0BS70ZZ<br>0BV44CZ<br>0BSB0ZZ<br>0BVB7DZ<br>0BV90DZ<br>0BV58DZ<br>0BVB7ZZ<br>0BN74ZZ<br>0BMB0ZZ<br>0BV74DZ<br>0BF87ZZ<br>0BFB3ZZ<br>0BUB4JZ<br>0BV77ZZ<br>0BU747Z<br>0BQ93ZZ<br>0BFB7ZZ<br>0BV94ZZ<br>0BU44KZ<br>0BUB47Z

© 2023 Kim KM et al. *JAMA Network Open.*

		0BV50DZ<br>0BU807Z<br>0BV90CZ<br>0BU54JZ<br>0BQ57ZZ<br>0BVB3DZ<br>0BU407Z<br>0BV54DZ<br>0BQ97ZZ<br>0BV73CZ<br>0BS30ZZ<br>0BF60ZZ<br>0BU70JZ<br>0BV47DZ<br>0BF33ZZ<br>0BF83ZZ<br>0BV44ZZ<br>0BV93CZ<br>0BV83CZ<br>0BV90ZZ<br>0BU647Z<br>0BN70ZZ<br>0BV53ZZ<br>0BNB0ZZ<br>0BF48ZZ<br>0BQB7ZZ<br>0BU44JZ<br>0BU94JZ<br>0BV34CZ<br>0BVB3CZ<br>0BV50ZZ<br>0BQ37ZZ<br>0BNB4ZZ<br>0BF50ZZ<br>0BF63ZZ<br>0BU30KZ<br>0BV84ZZ<br>0BM30ZZ<br>0BQ48ZZ<br>0BS50ZZ<br>0BU60KZ<br>0BVB0ZZ<br>0BQ50ZZ<br>0BM70ZZ<br>0BV74CZ<br>0BU907Z<br>0BN73ZZ<br>0BVB4DZ<br>0BU947Z<br>0BUB0KZ<br>0BV33CZ<br>0BV37ZZ<br>0BF74ZZ<br>0BFB4ZZ

© 2023 Kim KM et al. *JAMA Network Open.*

		0BV50CZ<br>0BU34JZ<br>0BV43CZ<br>0BN60ZZ<br>0BQ94ZZ<br>0BV30DZ<br>0BU40KZ<br>0BV94DZ<br>0BN30ZZ<br>0BN44ZZ<br>0BV44DZ<br>0BF57ZZ<br>0BU34KZ<br>0BN58ZZ<br>0BNB8ZZ<br>0BF80ZZ<br>0BQ44ZZ<br>0BUB07Z<br>0BU90JZ<br>0BV67ZZ<br>0BU94KZ<br>0BU50KZ<br>0BF88ZZ<br>0BM50ZZ<br>0BV57DZ<br>0BV43DZ<br>0BF84ZZ<br>0BV84DZ<br>0BQ33ZZ<br>0BQ54ZZ<br>0BQ68ZZ<br>0BU30JZ<br>0BQ38ZZ<br>0BF40ZZ<br>0BN97ZZ<br>0BU84KZ<br>0BV48DZ<br>0BQB4ZZ<br>0BN87ZZ<br>0BF78ZZ<br>0BU80JZ<br>0BF38ZZ<br>0BV87ZZ<br>0BS60ZZ<br>0BN53ZZ<br>0BU447Z<br>0BQ98ZZ<br>0BQ53ZZ<br>0BN40ZZ<br>0BU507Z<br>0BQ58ZZ<br>0BV40CZ<br>0BU607Z<br>0BU90KZ

© 2023 Kim KM et al. *JAMA Network Open.*

		0BF34ZZ<br>0BU347Z<br>0BV37DZ<br>0BV68ZZ<br>0BV93ZZ<br>0BN84ZZ<br>0BVB0DZ<br>0BQ60ZZ<br>0BQB0ZZ<br>0BU707Z<br>0BN93ZZ<br>0BN68ZZ<br>0BV80ZZ<br>0BV53DZ<br>0BS80ZZ<br>0BVB4ZZ<br>0BN34ZZ<br>0BU70KZ<br>0BV88ZZ<br>0BV78DZ<br>0BV67DZ<br>0BF90ZZ<br>0BU547Z<br>0BF68ZZ<br>0BS90ZZ<br>0BQ80ZZ<br>0BU50JZ<br>0BQ47ZZ<br>0BN90ZZ<br>0BU847Z<br>0BV63DZ<br>0BU40JZ<br>0BV48ZZ<br>0BNB3ZZ<br>0BQ67ZZ<br>0BS40ZZ<br>0BV38ZZ<br>0BF53ZZ<br>0BU54KZ<br>0BV47ZZ<br>0BF64ZZ<br>0BV84CZ<br>0BFB8ZZ<br>0BV60ZZ<br>0BV80CZ<br>0BN50ZZ<br>0BQ83ZZ<br>0BN47ZZ<br>0BQ40ZZ<br>0BV93DZ<br>0BV87DZ<br>0BQ63ZZ<br>0BQB3ZZ<br>0BU64KZ

© 2023 Kim KM et al. *JAMA Network Open.*

		0BQ73ZZ
		0BV78ZZ
		0BV74ZZ
		0BN78ZZ
		0BU74JZ
		0BQ43ZZ
		0BF98ZZ
		0BF73ZZ
		0BN63ZZ
		0BVB3ZZ
		0BV33ZZ
		0BF97ZZ
		0BQ77ZZ
		0BV97ZZ
		0BN38ZZ
		0BN57ZZ
		0BV64ZZ
		0BUB4KZ
		0BQ88ZZ
		0BN67ZZ
		0BV58ZZ
		0BF37ZZ
		0BQ64ZZ
		0BM40ZZ
		0BV88DZ
		0BQ30ZZ
		0BVB4CZ
		0BN48ZZ
		0BN83ZZ
		0BF70ZZ
		0BF43ZZ
		0BV60CZ
		0BN54ZZ
		0BV63CZ
		0BV57ZZ
		0BV80DZ
		0BQ87ZZ
		0BV83ZZ
		0BQ34ZZ
		0BV60DZ
		0BU64JZ
		0BV73ZZ
		0BVB8ZZ
		0BN98ZZ
		0BF44ZZ
		0BV70ZZ
		0BVB0CZ
		0BU307Z
		0BQ70ZZ
		0BM90ZZ
		0BF67ZZ
		0BF54ZZ
		0BN80ZZ
		0BN88ZZ

© 2023 Kim KM et al. *JAMA Network Open.*

				0BV64CZ
				0BV30CZ
				0BF93ZZ
				0BUB0JZ
				0BV34DZ
				0BV70DZ
				0BV98ZZ
				0BF47ZZ
				0BQB8ZZ
				0BN94ZZ
				0BU74KZ
				0BV34ZZ
				0BN43ZZ
				0BV30ZZ
				0BM80ZZ
				0BQ90ZZ
				0BF30ZZ
				0BFB0ZZ
				0BV40DZ
				0BN37ZZ
				0BQ84ZZ
				0BV73DZ
				0BV70CZ
				0BV54CZ
				0BF77ZZ
				0BF58ZZ
				0BN77ZZ
				0BV83DZ
				0BV38DZ
				0BN64ZZ
				0BQ74ZZ
				0BU80KZ
				0BM60ZZ
				0BN33ZZ
				0BV77DZ
				0BF94ZZ
				0BQ78ZZ
				0BV94CZ
				0BVB8DZ
				0BV97DZ
				0BU60JZ
				0BNB7ZZ
				0BV68DZ
				0BV33DZ
				0BV43ZZ
Thoracic	Extrapulmonary procedure	decortication of lung	34.51	0BDN0ZX
				0BDN3ZZ
				0BDN0ZZ
				0BDP3ZX
				0BDP0ZZ
				0BDN3ZX
				0BDP3ZZ
				0BDP0ZX
		other pleural excision	34.59	0BBP4ZZ

© 2023 Kim KM et al. *JAMA Network Open.*

				0BBN4ZZ
				0BBP3ZZ
				0BBP0ZZ
				0BBN0ZZ
				0BBN3ZZ
		clos thoracic fistul nec	34.73	0WQ84ZZ
				0WQ83ZZ
				0WQC4ZZ
				0BQ33ZZ
				0BQ80ZZ
				0BQ50ZZ
				0BQ97ZZ
				0BQ38ZZ
				0BQN3ZZ
				0BQ84ZZ
				0BQP0ZZ
				0BQ44ZZ
				0BQB3ZZ
				0BQN4ZZ
				0BQ83ZZ
				0BQ84ZZ
				0BQ83ZZ
				0BQ30ZZ
				0WQ80ZZ
				0WQ80ZZ
				0BQK7ZZ
				0BQ93ZZ
				0BQ48ZZ
				0BQ53ZZ
				0BQ33ZZ
				0FQ03ZZ
				0WQ84ZZ
				0BQ64ZZ
				0BQP3ZZ
				0BQP4ZZ
				0BQ88ZZ
				0BQB3ZZ
				0BQ33ZZ
				0BQP3ZZ
				0BQ53ZZ
				0WQ83ZZ
				0BQ50ZZ
				0BQ77ZZ
				0BQ68ZZ
				0BQP3ZZ
				0BQP4ZZ
				0BQ77ZZ
				0BQ67ZZ
				0WQC3ZZ
				0WQ84ZZ
				0BQB0ZZ
				0BQ73ZZ
				0BQ38ZZ
				0BQ43ZZ

© 2023 Kim KM et al. *JAMA Network Open.*

		0BQM3ZZ<br>0BQ78ZZ<br>0BQB0ZZ<br>0BQ97ZZ<br>0BQ57ZZ<br>0BQN3ZZ<br>0BQ30ZZ<br>0BQ88ZZ<br>0BQM4ZZ<br>0BQ63ZZ<br>0BQ53ZZ<br>0BQB0ZZ<br>0BQP0ZZ<br>0BQ38ZZ<br>0WQC0ZZ<br>0BQ68ZZ<br>0BQ70ZZ<br>0WQC0ZZ<br>0BQ48ZZ<br>0BQ44ZZ<br>0BQM8ZZ<br>0BQB4ZZ<br>0BQM0ZZ<br>0BQN4ZZ<br>0BQ54ZZ<br>0BQ94ZZ<br>0WQ83ZZ<br>0FQ00ZZ<br>0BQ43ZZ<br>0BQ64ZZ<br>0BQ98ZZ<br>0BQN4ZZ<br>0BQ30ZZ<br>0BQP0ZZ<br>0BQ37ZZ<br>0FQ00ZZ<br>0BQ63ZZ<br>0BQN3ZZ<br>0BQN0ZZ<br>0BQ77ZZ<br>0BQB8ZZ<br>0BQ90ZZ<br>0BQL7ZZ<br>0BQ68ZZ<br>0BQ78ZZ<br>0BQM7ZZ<br>0DQ53ZZ<br>0BQN3ZZ<br>0WQ80ZZ<br>0BQB4ZZ<br>0BQN0ZZ<br>0BQ84ZZ<br>0BQ34ZZ<br>0WQC4ZZ

		0BQK0ZZ
		0BQ37ZZ
		0BQN3ZZ
		0BQ63ZZ
		0BQ40ZZ
		0BQ33ZZ
		0BQP3ZZ
		0BQ84ZZ
		0BQ98ZZ
		0BQ94ZZ
		0BQ74ZZ
		0BQN4ZZ
		0BQ74ZZ
		0BQL4ZZ
		0BQB0ZZ
		0BQ97ZZ
		0BQ47ZZ
		0BQP4ZZ
		0BQ43ZZ
		0BQ94ZZ
		0BQ90ZZ
		0BQ74ZZ
		0WQC3ZZ
		0WQC4ZZ
		0BQN0ZZ
		0BQ47ZZ
		0BQ83ZZ
		0BQ93ZZ
		0BQK8ZZ
		0BQ88ZZ
		0BQB4ZZ
		0BQ40ZZ
		0BQ48ZZ
		0BQB7ZZ
		0BQ60ZZ
		0BQ44ZZ
		0BQ94ZZ
		0BQP0ZZ
		0BQ43ZZ
		0BQ60ZZ
		0BQ80ZZ
		0BQN3ZZ
		0BQ60ZZ
		0BQ67ZZ
		0BQ87ZZ
		0BQ68ZZ
		0BQ57ZZ
		0BQ73ZZ
		0BQ48ZZ
		0BQ50ZZ
		0BQ57ZZ
		0BQB7ZZ
		0BQ80ZZ
		0BQB4ZZ

© 2023 Kim KM et al. *JAMA Network Open.*

		0BQ98ZZ
		0WQ84ZZ
		0BQ70ZZ
		0BQ57ZZ
		0BQ93ZZ
		0BQ90ZZ
		0BQP0ZZ
		0BQ87ZZ
		0BQ97ZZ
		0BQ34ZZ
		0BQP4ZZ
		0BQ73ZZ
		0DQ58ZZ
		0BQ58ZZ
		0BQB3ZZ
		0BQB7ZZ
		0BQ64ZZ
		0BQ58ZZ
		0BQ78ZZ
		0BQP4ZZ
		0BQN4ZZ
		0BQN4ZZ
		0BQ90ZZ
		0BQ63ZZ
		0BQP4ZZ
		0BQ54ZZ
		0DQ54ZZ
		0BQ88ZZ
		0WQC3ZZ
		0FQ04ZZ
		0BQ40ZZ
		0BQB3ZZ
		0BQ70ZZ
		0BQ40ZZ
		0BQ98ZZ
		0BQL3ZZ
		0DQ57ZZ
		0BQ38ZZ
		0BQ80ZZ
		0BQ44ZZ
		0BQP3ZZ
		0BQ60ZZ
		0BQ87ZZ
		0BQ58ZZ
		0BQ34ZZ
		0BQ78ZZ
		0BQL8ZZ
		0BQK4ZZ
		0BQ73ZZ
		0BQB8ZZ
		0BQB8ZZ
		0BQ54ZZ
		0BQ50ZZ
		0WQ83ZZ

				0BQN0ZZ<br>0BQ74ZZ<br>0BQ54ZZ<br>0FQ03ZZ<br>0BQ67ZZ<br>0BQ64ZZ<br>0BQ37ZZ<br>0BQP3ZZ<br>0BQ70ZZ<br>0BQB7ZZ<br>0BQL0ZZ<br>0BQB8ZZ<br>0WQC0ZZ<br>0DQ50ZZ<br>0BQ37ZZ<br>0BQP0ZZ<br>0BQ47ZZ<br>0BQ34ZZ<br>0BQ53ZZ<br>0BQ30ZZ<br>0BQ87ZZ<br>0BQ93ZZ<br>0BQ67ZZ<br>0BQN0ZZ<br>0WQ80ZZ<br>0BQ47ZZ<br>0BQ58ZZ<br>0BQ83ZZ<br>0BQN0ZZ<br>0FQ04ZZ<br>0BQK3ZZ<br>0BQ77ZZ
Cardiac	Mediastinal procedure	incision of mediastinum	34.1	0W9C00Z<br>0W9C0ZZ<br>0W9C30Z<br>0W9C3ZZ<br>0W9C40Z<br>0W9C4ZZ<br>0WCC0ZZ<br>0WCC3ZZ<br>0WCC4ZZ
Cardiac	Heart valve procedures	opn aortic valvuloplasty	35.11	02QF0ZZ<br>027F0ZZ<br>02NF0ZZ<br>027F0DZ<br>027F04Z
		opn mitral valvuloplasty	35.12	02QG0ZZ<br>027G04Z<br>027G0DZ<br>027G0ZZ<br>02NG0ZZ
		opn tricus valvuloplasty	35.14	027J0DZ<br>02NJ0ZZ<br>027J04Z

© 2023 Kim KM et al. *JAMA Network Open.*

				027J0ZZ<br>02QJ0ZZ
		replace aort valv-tissue	35.21	02RF08Z<br>02RF07Z<br>02RF0KZ<br>02RF4KZ<br>02RF47Z<br>02RF48Z
		replace aortic valve nec	35.22	02RF0JZ<br>02RF4JZ
		replace mitr valv-tissue	35.23	02RG3KZ<br>02RG07Z<br>02RG08Z<br>02RG48Z<br>02RG47Z<br>02RG0KZ<br>02RG37Z<br>02RG38Z<br>02RG4KZ
		replace mitral valve nec	35.24	02RG4JZ<br>02RG0JZ<br>02RG3JZ
		replace tric valv-tissue	35.27	02RJ48Z<br>02RJ4KZ<br>02RJ08Z<br>02RJ07Z<br>02RJ0KZ<br>02RJ47Z
		replace tricusp valv nec	35.28	02RJ0JZ<br>02RJ4JZ
Cardiac	Coronary artery bypass graft (CABG)	aortocor bypas-1 cor art	36.11	021009W<br>02100KW<br>02104AW<br>02104KW<br>021049W<br>02104JW<br>02100AW<br>02100JW
		aortocor bypas-2 cor art	36.12	021149W<br>02110KW<br>021109W<br>02114JW<br>02110JW<br>02110AW<br>02114KW<br>02114AW
		aortocor bypas-3 cor art	36.13	021249W<br>02124KW<br>02124JW<br>02120JW<br>02120AW<br>02120KW<br>02124AW

© 2023 Kim KM et al. *JAMA Network Open.*

				021209W
		aortcor bypas-4+ cor art	36.14	02130AW
				02130JW
				02130KW
				021349W
				02134JW
				02134KW
				021309W
				02134AW
		1 int mam-cor art bypass	36.15	02100JC
				02100J8
				02100A8
				02104JC
				02104A9
				0210099
				02100KC
				02104K9
				0210498
				02104K8
				02104Z9
				021049C
				02104A8
				02100K9
				02100AC
				02100K8
				02104KC
				02100Z9
				02104Z8
				02104J8
				02100ZC
				0210499
				02100J9
				02104J9
				02100Z8
				02100A9
				0210098
				02104AC
				021009C
				02104ZC
		2 int mam-cor art bypass	36.16	02114AC
				02114KC
				02110J8
				02124AC
				02110AC
				02130ZC
				02134ZC
				0211098
				02134JC
				0211498
				02114J9
				021349C
				0211499
				02124JC
				02114K9

© 2023 Kim KM et al. *JAMA Network Open.*

				0211099
				02114Z9
				02110Z8
				021109C
				02114JC
				02130JC
				02110ZC
				02134KC
				02114ZC
				02120KC
				021309C
				02124ZC
				02120JC
				02110KC
				02130AC
				02114A9
				02130KC
				02120ZC
				02110A9
				02110A8
				02134AC
				021249C
				02114K8
				02110J9
				02114Z8
				02124KC
				02110Z9
				02110JC
				02114J8
				021209C
				02114A8
				02120AC
				021149C
				02110K9
				02110K8
		open chest trans revasc	36.31	021L0Z5
				021K0Z5
	Pericardial/Extracardiac	heart & pericard repair#	37.40	
		pericardiotomy	37.12	0W9D0ZZ
				02NN3ZZ
				0WCD4ZZ
				02NN0ZZ
				02CN4ZZ
				02CN0ZZ
				02NN4ZZ
				0W9D0ZX
				0W9D00Z
				0WCD0ZZ
				0WCD3ZZ
				02CN3ZZ
		pericardiectomy	37.31	02BN3ZZ
				02TN4ZZ
				02BN0ZZ
				02TN0ZZ

© 2023 Kim KM et al. *JAMA Network Open.*

				02BN4ZZ<br>02TN3ZZ
Cardiac	Intracardiac	annuloplasty	35.33	02QJ0ZZ<br>02QG3ZZ<br>02QH0ZZ<br>02QH4ZZ<br>02QF4ZZ<br>02QJ4ZZ<br>02QH3ZZ<br>02QJ3ZZ<br>02QG4ZZ<br>02QF0ZZ<br>02QG0ZZ<br>02QF3ZZ
		pros rep ventric def-opn	35.53	02UM4JZ<br>02UM3JZ<br>02RM0JZ<br>02UM0JZ
		graft repair ventric def	35.62	02UM07Z<br>02RM07Z<br>02UM38Z<br>02RM0KZ<br>02RM47Z<br>02UM48Z<br>02UM0KZ<br>02RM4KZ
		ventr septa def rep nec	35.72	02QM4ZZ<br>02QM0ZZ<br>02QM4ZZ<br>02QM3ZZ
		conduit left ventr-aorta	35.93	021L0ZW<br>021L4ZW
		cardiotomy	37.11	02CK3ZZ<br>02CL3ZZ<br>02C60ZZ<br>02C84ZZ<br>02CL4ZZ<br>02C93ZZ<br>02C90ZZ<br>02CK0ZZ<br>02C83ZZ<br>02CL0ZZ<br>02C73ZZ<br>02C80ZZ<br>02C70ZZ<br>02CK4ZZ<br>02C74ZZ<br>02C63ZZ<br>02C64ZZ<br>02C94ZZ
		heart aneurysm excision	37.32	02B64ZZ<br>02B73ZZ<br>02BL3ZZ<br>02B60ZZ

© 2023 Kim KM et al. *JAMA Network Open.*

				02BK3ZZ
				02BL4ZZ
				02BK4ZZ
				02BL0ZZ
				02B63ZZ
				02BK0ZZ
				02B70ZZ
				02B74ZZ
		exc/dest hrt lesion open	37.33	02B70ZZ
				025L0ZZ
				02560ZZ
				02570ZZ
				025K0ZZ
				02BK0ZZ
				02T80ZZ
				02B60ZZ
				02BL0ZZ
		partial ventriculectomy	37.35	02BL3ZZ
				02BK3ZZ
				02BK0ZZ
				02BL4ZZ
				02BK4ZZ
				02BL0ZZ
Cardiac	Other Coronary Artery procedure	open coronry angioplasty	36.03	02C10ZZ
				02730ZZ
				02720ZZ
				02C20ZZ
				02C30ZZ
				02710ZZ
				02700ZZ
				02C00ZZ
		rem of cor art obstr nec	36.09	02C13ZZ
				02C33ZZ
				02C03ZZ
				02C04ZZ
				02C14ZZ
				02C23ZZ
				02C34ZZ
				02C24ZZ
		heart vessel op nec	36.99	02Q00ZZ
				02Q04ZZ
				02Q43ZZ
				02Q44ZZ
				02Q40ZZ
				02Q03ZZ
Cardiac	Artificial Heart/Assist Devices	insrt non-impl circ dev	37.62	5A02216
				5A02116
		repair heart assist sys	37.63	02WA3QZ
				02WA0RZ
				02WA3RZ
				02WA4QZ
				02WA4RZ
				02WA0QZ

© 2023 Kim KM et al. *JAMA Network Open.*

		imp vent ext hrt ast sys	37.65	02HA0RZ
		implantable hrt assist	37.66	02HA3QZ
				02HA0QZ
				02HA4QZ
				02HA3QZ
				02HA0QZ
				02HA4QZ
				5A02216
				5A02116
Cardiac	Aortic resection; replacement of anastomosis	aorta resection & anast	38.34	02BW4ZZ
				02BW0ZZ
				04B00ZZ
				04B04ZZ
		resect abdm aorta w repl	38.44	04R00JZ
				04R04JZ
				04R047Z
				04R007Z
				04R04KZ
				04R00KZ
		excision of aorta	38.64	04B03ZZ
				04503ZZ
				04504ZZ
				04B04ZZ
				04B00ZZ
				04500ZZ
		endo imp grft thor aorta	39.73	02VW4DZ
				02VW0DZ
				02UW3JZ
				02UW4JZ
				02VW3DZ
Cardiac	Peripheral Vascular Bypass	aorta-iliac-femor bypass	39.25	041C0JK
				041J4JH
				04104JR
				041D4ZH
				041F0ZK
				041C0KH
				041H09J
				041F0JJ
				041049Q
				04100JB
				041F0ZJ
				041C4JH
				04100ZH
				04104A9
				041E0KK
				041E0AJ
				041D09K
				041E0ZJ
				04100A6
				041F4AH
				041J4KK
				041C4JK
				041H49H

		041E0JJ<br>041E49J<br>041D0KK<br>041F4JK<br>041C09H<br>041E4ZK<br>041D0JH<br>041H0AH<br>0410096<br>041J4JJ<br>041009H<br>04104AB<br>04100ZF<br>041E09H<br>04100AQ<br>04100ZB<br>041C4ZH<br>04100KC<br>04100AK<br>041H49J<br>04104ZG<br>041D0AJ<br>041J4JK<br>041J0JJ<br>04104K8<br>04104ZR<br>04100K9<br>041H0AJ<br>041F4ZH<br>041J09K<br>041H4AK<br>041H4ZK<br>0410496<br>041F0AH<br>041F09K<br>041H4ZH<br>04100JF<br>041E4AK<br>041C0ZK<br>04104JQ<br>041F0JK<br>041D09H<br>0410499<br>04100KD<br>041H4AJ<br>041C0AJ<br>041H0ZK<br>041D0ZJ<br>04104A6<br>041J4ZH<br>04104KC<br>04104ZC<br>04100JC<br>041E0KJ

© 2023 Kim KM et al. *JAMA Network Open.*

		041D49J<br>041H09K<br>041H0ZJ<br>041E0ZK<br>041H0KK<br>041E4AJ<br>041H0JJ<br>041C09J<br>041H4KH<br>041009G<br>04100JD<br>04104Z8<br>041E4JK<br>041D0AK<br>041D4ZJ<br>041D09J<br>041C09K<br>041009J<br>04100AB<br>041J0JK<br>04104A8<br>04100JQ<br>041C49H<br>04104J8<br>041C4ZK<br>04104J9<br>04100ZJ<br>041049F<br>041F0KK<br>041H4AH<br>04104A7<br>04104K6<br>041C4AJ<br>04100JH<br>04100A9<br>04100JJ<br>041J0KJ<br>041009D<br>041J49K<br>041D49K<br>04104Z9<br>041J0AJ<br>04104KJ<br>041C0AH<br>041C0ZH<br>041D0AH<br>041F0AJ<br>041F4ZJ<br>04100KF<br>041E49K<br>04100AJ<br>04104KF<br>041E0ZH<br>041H4KK

© 2023 Kim KM et al. *JAMA Network Open.*

		041F09J
		04104JB
		041J4ZK
		041D0ZH
		041J0JH
		041049C
		04104ZJ
		04100J7
		04100J9
		041D0JJ
		041J0ZH
		041C0JJ
		041049D
		04100KH
		04104AK
		041H4ZJ
		041J0ZJ
		04104KR
		041049B
		04104ZB
		041C49K
		041F49J
		04100ZG
		041D4JK
		04100KQ
		04100KK
		04104ZH
		04104K9
		04104JD
		041J4AJ
		04104JK
		04100K7
		04104AH
		04100AC
		041J0KH
		04100KR
		041C0ZJ
		04104KD
		041E4AH
		04104AR
		041049R
		041D0ZK
		041009B
		041H09H
		04104KQ
		041F4ZK
		041J0ZK
		04100Z7
		04104Z7
		04100KJ
		04104J6
		041C4ZJ
		041E4KK
		041E4ZH

		041E4KH<br>0410097<br>04100JR<br>041J4KH<br>041D4KJ<br>041D0KH<br>04104KK<br>04100AR<br>04100ZC<br>04104JC<br>041E4KJ<br>041C0KK<br>041H0AK<br>04100J6<br>041F0JH<br>041H4JH<br>041E4JJ<br>041D0KJ<br>041C4AK<br>04104JG<br>04100ZD<br>041D4KH<br>041E4JH<br>041F0KJ<br>041J4ZJ<br>041E0JH<br>04100KG<br>04100K6<br>041009K<br>04104KH<br>04104J7<br>04100ZQ<br>041E09K<br>041C0JH<br>041F09H<br>04104AD<br>041J4KJ<br>041C0AK<br>041J49J<br>041J09H<br>041H4JJ<br>041D0JK<br>041J0KK<br>041H0KH<br>041J4AH<br>04100Z6<br>04100AD<br>04100Z8<br>041C0KJ<br>041J09J<br>041D4KK<br>041C4AH<br>04104JH<br>04100Z9

© 2023 Kim KM et al. *JAMA Network Open.*

		041009Q
		041E0AK
		041C4KJ
		041H49K
		041H4KJ
		041F49K
		04100KB
		04104JJ
		041D4JJ
		04104K7
		04100J8
		041J49H
		041049J
		041E49H
		041009R
		041D4AH
		04100AH
		041H0KJ
		041C4KK
		04104ZQ
		04104ZD
		041D4AK
		0410498
		04100A7
		0410098
		041F0ZH
		041H4JK
		04100ZK
		041D4ZK
		041H0JH
		041E4ZJ
		041049K
		041D49H
		041E0AH
		041F4JH
		04100ZR
		041F4AJ
		04104ZK
		041009F
		04104AF
		041J0AH
		041D4AJ
		04104KB
		041F4AK
		04104AC
		041H0JK
		04104AQ
		04104AJ
		0410099
		041009C
		041D4JH
		041J0AK
		041F4JJ
		04100K8

© 2023 Kim KM et al. *JAMA Network Open.*

				041049H
				041F0KH
				041F4KK
				04104JF
				04104KG
				041C49J
				041E09J
				04100A8
				041C4KH
				041C4JJ
				04100JK
				04100JG
				04104Z6
				041049G
				041J4AK
				041F0AK
				041F49H
				04100AF
				041E0KH
				041F4KH
				041H0ZH
				0410497
				041E0JK
				041F4KJ
				04104AG
				04100AG
				04104ZF
		vasc shunt & bypass nec	39.29	051G0KY
				041K0KL
				0313097
				0316090
				03150JF
				061V4JY
				041K09S
				031A0ZF
				051A07Y
				0316098
				03190KF
				051N4JY
				031209B
				03130Z6
				03150A8
				03140J3
				051T0ZY
				031C0AF
				041L0KS
				051M47Y
				03140A9
				041K09L
				041K0ZH
				051G07Y
				03130A0
				03120KC
				03160J5

© 2023 Kim KM et al. *JAMA Network Open.*

		03150ZJ<br>03140K9<br>051V0ZY<br>051L0AY<br>061N4KY<br>051H47Y<br>031B093<br>03130K6<br>031609C<br>061M0AY<br>061M0JY<br>031B09F<br>041L0AJ<br>041K0AN<br>061F0JY<br>03160AJ<br>03140ZK<br>041K4JL<br>061C07Y<br>061G09Y<br>03140KC<br>061309Y<br>061R4JY<br>041K0AL<br>031J0AG<br>051G4KY<br>03150ZF<br>061Q0JY<br>041K4JK<br>03120KJ<br>03120AD<br>031609D<br>03130KB<br>03130K2<br>031G0AG<br>051F4JY<br>03150J3<br>03150KC<br>03120ZJ<br>031A09F<br>03120Z0<br>03130Z1<br>03140J9<br>051G0AY<br>051S07Y<br>03150Z3<br>03160A6<br>041L4KM<br>03130K0<br>03160J6<br>03130A7<br>041L4AQ<br>05174KY<br>041K4ZL

© 2023 Kim KM et al. *JAMA Network Open.*

		041N4JQ
		03160Z0
		03170JF
		041L0JM
		06130AY
		03170JD
		041N4KS
		03140JC
		031A0J4
		03160ZC
		051V0AY
		061V4AY
		041K0AM
		051V0JY
		05180JY
		031J0KG
		061P07Y
		0314090
		041L0JK
		041L09Q
		041K4ZN
		031H0JG
		041M4AM
		061R0AY
		03160K2
		061F4AY
		051R0KY
		041M0JS
		051T49Y
		051P4KY
		03190Z3
		03160AC
		03140JF
		05174JY
		03150KD
		061G0KY
		051R4JY
		06130ZY
		06134KY
		0313091
		03120ZK
		051Q07Y
		03120Z1
		041K0JN
		051H0JY
		051B0ZY
		051T0AY
		03160K9
		061G4JY
		041K4KH
		061M4ZY
		03180JD
		061S49Y
		051C07Y

		031A0K4<br>051V4ZY<br>041N0JL<br>05184JY<br>041K09Q<br>031809F<br>061R47Y<br>03160Z4<br>0316095<br>041K4AJ<br>051S49Y<br>03160Z2<br>03160K4<br>051F0ZY<br>03140Z7<br>061S0KY<br>061Q09Y<br>051L0KY<br>041L09H<br>041L0JJ<br>03170Z0<br>03130K1<br>05194ZY<br>041K49M<br>041M49L<br>041M0KP<br>041M4AQ<br>03160J8<br>051C4JY<br>041N4ZS<br>03150A7<br>041K4ZH<br>061H07Y<br>03130JC<br>041K0AK<br>03160J2<br>061V4ZY<br>041L4JS<br>03160JD<br>041L4JH<br>03150J7<br>061F0KY<br>041K0AS<br>03140A1<br>061M4AY<br>03120K3<br>061T4ZY<br>03160ZF<br>06134ZY<br>061S09Y<br>041L49K<br>061N0KY<br>061S4AY<br>03130J9

© 2023 Kim KM et al. *JAMA Network Open.*

		051H09Y
		041N4ZM
		061R0ZY
		03180K4
		0312093
		041K4JJ
		03130A4
		051A4AY
		03140KJ
		051Q0ZY
		0318091
		03130AJ
		061C47Y
		061H0AY
		041L09S
		041M0JM
		061M4KY
		041K4JN
		041M0ZP
		051A09Y
		041N0AS
		03120JF
		051G47Y
		031809D
		031J0ZG
		051P09Y
		03140J7
		061T0KY
		03160A5
		03130J6
		03160K0
		061P0KY
		03130Z2
		061347Y
		03150K3
		03150A4
		03120JB
		041K09J
		051A49Y
		031G0JG
		03120Z9
		03170A3
		03120K1
		061P49Y
		041K0KM
		061G47Y
		03140A8
		051D09Y
		031G09G
		041L0JP
		031A0JF
		03130AF
		061G07Y
		03160A0

		031C09F<br>061N47Y<br>041L0AM<br>03120A7<br>041L49H<br>03130AK<br>031B0A3<br>0315096<br>05190KY<br>061C09Y<br>041K0ZJ<br>031C0ZF<br>051G09Y<br>03160KD<br>041K0KQ<br>061C0ZY<br>041N4AQ<br>03170ZF<br>03150AJ<br>041K49P<br>03160JF<br>051S4JY<br>041K4KQ<br>03140JD<br>051F0KY<br>03120A8<br>03120A3<br>03160Z9<br>051C49Y<br>061H0JY<br>041M4JS<br>03120KB<br>041N0ZL<br>051G0JY<br>041L09J<br>031C0A4<br>03150AD<br>0315092<br>041M49P<br>03160J4<br>061N0ZY<br>041M0ZL<br>041K0ZQ<br>051847Y<br>03150K7<br>031C0J4<br>061Q4KY<br>041K4KN<br>061Q0ZY<br>041N49P<br>041K4AH<br>05184AY<br>041N09P<br>041M09S

© 2023 Kim KM et al. *JAMA Network Open.*

		05170KY
		03140JK
		03120KD
		03160JC
		051H07Y
		041K4KS
		03160AB
		031509C
		051V4AY
		03130A1
		041N0KL
		051C47Y
		061Q4AY
		03120K0
		06134JY
		0312095
		031A0A4
		0315090
		031609B
		03140J1
		041M0ZQ
		041K0AJ
		051P4ZY
		03180ZD
		03150J1
		05194AY
		031C0KF
		051A47Y
		061V09Y
		03130K7
		051S4AY
		03150K9
		051P0JY
		03140AF
		051M49Y
		051M0ZY
		061N09Y
		03120K4
		041M4ZP
		041L4KP
		05190AY
		051T0JY
		061N4AY
		051G4ZY
		051G49Y
		051907Y
		061H4JY
		031209J
		03120J3
		051R07Y
		041M0KQ
		041K4KM
		03160Z6
		041N49S

		041L4KS
		041M4KQ
		03130ZK
		031H0ZG
		041M4KS
		03150JJ
		0315095
		03140K8
		061N49Y
		03120K9
		0316091
		041M09L
		041L0JQ
		041N09M
		051N4AY
		051R0JY
		0312091
		041M09Q
		0314094
		03120A0
		061C49Y
		041N4KP
		041M4JM
		041K49S
		03130Z5
		03130ZJ
		061D0ZY
		041M4KL
		0314099
		031B0ZF
		051B09Y
		03140K5
		051D0KY
		051S0JY
		061P09Y
		041K0KS
		031209K
		061M49Y
		03120Z6
		041N0ZQ
		031C0JF
		041K0KK
		051C09Y
		03130Z8
		03150K2
		031A0KF
		041M0JQ
		061P0ZY
		031209D
		041K4AP
		03180J4
		03120J1
		03170ZD
		051M09Y

© 2023 Kim KM et al. *JAMA Network Open.*

		03150J8
		03130AB
		041N4ZL
		03130JB
		03120Z4
		051707Y
		03130AD
		061V0JY
		041N49M
		03140ZF
		041M4ZQ
		03150Z4
		061S47Y
		061D09Y
		041K4ZS
		041K4JM
		03120A4
		0312092
		041L0ZL
		03160A9
		03160A2
		041K4KJ
		03140A3
		0314095
		051P49Y
		041L4ZP
		051M0KY
		061G0JY
		03150A6
		041L09P
		061Q49Y
		03150A5
		031609K
		041K4AL
		0316096
		051C0JY
		061V0KY
		03140JJ
		031509B
		051M0JY
		03150Z7
		061C0JY
		05170AY
		061M0ZY
		061N4ZY
		03130ZB
		031C0K4
		061V4KY
		041K0ZK
		061R4AY
		03130Z3
		051N09Y
		061F07Y
		041K0ZN

© 2023 Kim KM et al. *JAMA Network Open.*

		03140KK
		03120JD
		041M4AL
		03170Z3
		03130A9
		0317090
		03160J7
		051P07Y
		051B0KY
		03150JB
		051N0KY
		051D4JY
		031509D
		051S0KY
		03140J4
		061V07Y
		051M4ZY
		03130J3
		041M0AQ
		041L0KP
		03120ZF
		041K0KP
		061T0ZY
		03150ZD
		03130KJ
		041K0JK
		051B07Y
		061D47Y
		041L4JP
		03150K5
		051M4AY
		051S4ZY
		051B4AY
		051V07Y
		041M0KM
		041L4KJ
		03190A3
		03160K5
		051F49Y
		041M4JQ
		0316092
		051A0JY
		061R0JY
		0315097
		03180K1
		051V0KY
		03140A6
		041K0KJ
		03160A3
		061T47Y
		03140Z6
		041L49L
		0312098
		061S4ZY

© 2023 Kim KM et al. *JAMA Network Open.*

		03160Z3
		051949Y
		061T4KY
		03180A1
		061N0AY
		03150K8
		0315099
		031B0K3
		051B4ZY
		061M09Y
		051D0JY
		03150AK
		03120ZD
		03150JK
		051Q0KY
		061H0ZY
		041L0ZS
		03140AC
		03150AB
		041N0KS
		03120AJ
		041L4ZQ
		03130Z0
		03150Z5
		03130KD
		041K4ZM
		03120J9
		051T09Y
		051L4KY
		041L4JL
		041M49M
		03140Z8
		051H4ZY
		03150J2
		0316094
		03150ZC
		061C4ZY
		061F49Y
		051P0KY
		03140Z4
		041N09Q
		061H47Y
		051A0KY
		0319093
		031G0KG
		03190AF
		0317093
		051Q47Y
		03160AF
		051B47Y
		03150A3
		051C4AY
		041K0AQ
		061C0AY

		03120ZC
		051L0JY
		03160ZJ
		041L4AL
		051H4JY
		03120J4
		03160KF
		03140Z1
		061T09Y
		041K0JP
		041L09M
		03140ZJ
		0313096
		051L4ZY
		041L09K
		061G4KY
		041L4JM
		041N0JP
		041L4AP
		041L0ZJ
		051N47Y
		03130K3
		051M07Y
		031C094
		041L0ZQ
		041N09L
		041K4AQ
		041L0JH
		061T0JY
		051M0AY
		051S47Y
		03120Z2
		03130KK
		031B0AF
		05170JY
		0312099
		031309C
		03150Z6
		041L0AS
		051D49Y
		041M0KL
		051A4KY
		03150ZK
		061H0KY
		061S4JY
		03120K8
		041L0KJ
		051849Y
		031G0ZG
		051N0ZY
		051F07Y
		061T0AY
		03160J0
		03140KD

© 2023 Kim KM et al. *JAMA Network Open.*

		061C0KY<br>03160K6<br>061H4KY<br>041L49N<br>061D4ZY<br>041M4KM<br>041L0KH<br>03180AD<br>041L0KL<br>051A0AY<br>031B0Z3<br>061Q4JY<br>03170A0<br>061M4JY<br>03140Z2<br>03160AD<br>03160J1<br>041N0ZP<br>051L07Y<br>03120AB<br>0312096<br>03140K0<br>03150ZB<br>03150Z1<br>061S4KY<br>041L49P<br>03140KB<br>061Q0KY<br>061M0KY<br>041L0JL<br>041N0AL<br>051C4ZY<br>03130A6<br>03130K5<br>051L4AY<br>03140ZB<br>041K0AP<br>031509F<br>061R0KY<br>03140Z9<br>061P4ZY<br>0315091<br>03120J5<br>041L4JN<br>03150J5<br>03140J8<br>051V49Y<br>03140J2<br>051R0ZY<br>041M0AL<br>03120K7<br>051H0KY<br>03190JF<br>051B4KY

		041L49J
		03130ZD
		041K4ZQ
		03180J1
		041L4ZH
		041K09P
		03150J6
		03160A8
		041L4ZS
		0315093
		041L4AM
		051A4JY
		061C4KY
		0314097
		03160JB
		041K49H
		031409F
		03160A7
		051809Y
		031609J
		051Q49Y
		03130Z4
		051Q4JY
		05190JY
		061S0JY
		041M49S
		03120ZB
		061H49Y
		031H0AG
		041L0AQ
		051Q4KY
		03140AK
		061F0AY
		03130A2
		051A0ZY
		041M4JL
		051D4ZY
		03130ZC
		031309K
		031209F
		03140A0
		03120J0
		03160ZD
		031B0KF
		061G0ZY
		031409B
		03120A6
		041K0JJ
		03160A1
		041K4JP
		061D4JY
		041K0JH
		031B0JF
		03140K1

© 2023 Kim KM et al. *JAMA Network Open.*

		03120A1
		03150K0
		041K49K
		041N4JL
		061G4AY
		031909F
		03170J0
		051T4JY
		041L0KK
		041K4KK
		041N4AP
		03120J8
		051R47Y
		051D4KY
		041K49L
		041L0ZH
		041M0ZM
		03120A5
		0313090
		0318094
		041L4AS
		03160J9
		051M4KY
		051C0ZY
		03170AD
		0313099
		03140A2
		03140AJ
		03120K6
		051V09Y
		03130A5
		041K4AK
		051R4AY
		051Q0AY
		05180ZY
		041M4JP
		05194JY
		041N49Q
		03140K6
		03160Z1
		061R49Y
		031H0KG
		031309D
		041M4AS
		041N4JP
		03160KC
		041K09M
		03130KC
		061N0JY
		041K09K
		041N0ZM
		03140Z3
		031509J
		051807Y

© 2023 Kim KM et al. *JAMA Network Open.*

		061D4KY
		03150AF
		041N0JQ
		051T4ZY
		051V47Y
		041L0ZM
		03130K8
		061D07Y
		05184KY
		041N4KQ
		03180A4
		051N07Y
		051A4ZY
		03150J0
		03140ZC
		061P0AY
		051F0AY
		061F09Y
		041N4JS
		03180KD
		041M0AM
		041L4KQ
		03180KF
		031209C
		031H09G
		03140Z0
		061V47Y
		061M07Y
		061P4AY
		051G4JY
		051P47Y
		041L4KK
		041M0KS
		03160Z5
		041K4ZJ
		041N0AM
		031A0Z4
		0316093
		03120Z7
		051N0AY
		03150KJ
		041L0KM
		03130A8
		051R4ZY
		03120A9
		03120Z3
		061T4AY
		03160J3
		041M4ZM
		03140JB
		041M0JL
		03150KF
		051G4AY
		03120AF

© 2023 Kim KM et al. *JAMA Network Open.*

		041L0JS<br>041M4AP<br>051P0AY<br>041M4ZS<br>041L4KN<br>03130A3<br>061R4ZY<br>051S09Y<br>03140Z5<br>051F4KY<br>051P0ZY<br>051F09Y<br>051T4KY<br>031C0Z4<br>05194KY<br>03150KK<br>051S0ZY<br>061C4JY<br>051L09Y<br>05180AY<br>0313098<br>061P4JY<br>041L4AJ<br>041K0JL<br>041N4KM<br>03150Z2<br>03160K3<br>041L0ZN<br>051F4ZY<br>051R49Y<br>061T07Y<br>03170K0<br>051749Y<br>041L09N<br>051N0JY<br>041N0JM<br>03160KB<br>03120J6<br>061S07Y<br>03120A2<br>041M0JP<br>03130J1<br>041M0AS<br>041K4AN<br>041N0KP<br>03130J8<br>03160JK<br>03120JJ<br>061P0JY<br>061D0AY<br>061D4AY<br>051Q4AY<br>051Q09Y<br>03120KF

© 2023 Kim KM et al. *JAMA Network Open.*

		03140K4<br>03120KK<br>051947Y<br>051F0JY<br>061R4KY<br>03160Z7<br>05180KY<br>03160K1<br>061T49Y<br>03130KF<br>041K4JH<br>03180ZF<br>061Q07Y<br>061G49Y<br>041N4AL<br>03180AF<br>0313092<br>0314091<br>03150K6<br>03150J9<br>051H0ZY<br>03120J2<br>0314092<br>03150K4<br>041N0AP<br>03150Z0<br>051L47Y<br>051N4ZY<br>03130ZF<br>03140J5<br>03140K3<br>051H49Y<br>03160ZB<br>041K0KN<br>031309B<br>041M0AP<br>041L4AH<br>041L49M<br>041L4KL<br>03140J0<br>041L0AK<br>051B0JY<br>061C4AY<br>041K0ZP<br>03150A0<br>05190ZY<br>061R09Y<br>051R4KY<br>03150JD<br>041K49N<br>061F0ZY<br>061D49Y<br>041L4ZM<br>041N0ZS

		061349Y<br>051F4AY<br>03130Z7<br>041L49S<br>041K4AS<br>03140KF<br>03160A4<br>041K4KL<br>031309F<br>03130J2<br>051L0ZY<br>0313094<br>051H4KY<br>051909Y<br>06130JY<br>041M09M<br>051B4JY<br>03120K2<br>051T0KY<br>03130J0<br>031509K<br>041N4AS<br>041N0AQ<br>03160Z8<br>061H4ZY<br>061Q4ZY<br>041M09P<br>0313095<br>041L49Q<br>061R07Y<br>0315098<br>03140K7<br>05184ZY<br>03180JF<br>051H0AY<br>041K09H<br>061S0AY<br>051D07Y<br>041L4JK<br>051M4JY<br>041L0ZK<br>051C0AY<br>041N4AM<br>061Q0AY<br>031409D<br>051B0AY<br>051D0ZY<br>03150Z9<br>041L4AN<br>051H4AY<br>061P4KY<br>03160JJ<br>031409C<br>041K4AM

© 2023 Kim KM et al. *JAMA Network Open.*

		03150A1
		031709D
		03130JJ
		03160KJ
		0316097
		05170ZY
		041N0KQ
		061F47Y
		041L0AN
		041K09N
		I029
		03120JC
		0314098
		051C0KY
		051L49Y
		061F4ZY
		03120Z8
		0316099
		041L4ZN
		03190J3
		03140AB
		061G4ZY
		061D0KY
		041M0ZS
		03120AC
		0314096
		061N07Y
		061V0AY
		031409J
		051S4KY
		041L4ZK
		03160ZK
		051F47Y
		051B49Y
		051T07Y
		03140A7
		061F4JY
		061P47Y
		061M47Y
		03130JK
		051709Y
		0313093
		03130JF
		041L4ZJ
		041L0JN
		03150JC
		041K0ZS
		031409K
		03140A4
		041L4AK
		03130J4
		03130Z9
		041N49L
		041L4ZL

© 2023 Kim KM et al. *JAMA Network Open.*

		05174ZY
		041L0AP
		051D0AY
		03170KF
		051Q0JY
		051T47Y
		041M4KP
		03120JK
		03180Z1
		041L09L
		03130K4
		03140K2
		041L0AL
		041L4JQ
		061Q47Y
		03160K7
		061307Y
		041N4ZQ
		05174AY
		041L0ZP
		03140AD
		031A0AF
		03130J5
		031J09G
		051P4JY
		03150A9
		061V0ZY
		051R09Y
		051747Y
		03130K9
		061F4KY
		051T4AY
		03190ZF
		031B0J3
		03170K3
		03150Z8
		03120J7
		041N4JM
		041N09S
		061H09Y
		03170J3
		041K49J
		03170AF
		03150AC
		041L0KN
		03140J6
		041K4ZK
		03130JD
		041N4KL
		041K4JS
		03160K8
		061D0JY
		03120Z5
		041K4KP

		041K0ZL<br>03140A5<br>0314093<br>051R0AY<br>051N4KY<br>06134AY<br>061G0AY<br>03150KB<br>061H4AY<br>031A094<br>051D47Y<br>041K0JM<br>03120K5<br>031309J<br>041K0KH<br>03160KK<br>031609F<br>041K0ZM<br>041K49Q<br>03120AK<br>03190K3<br>061N4JY<br>051D4AY<br>051V4JY<br>03130AC<br>051P4AY<br>03160AK<br>031J0JG<br>051Q4ZY<br>051L4JY<br>051N49Y<br>041M49Q<br>03180Z4<br>061S0ZY<br>051C4KY<br>041N0KM<br>051S0AY<br>041L4JJ<br>03130J7<br>0312090<br>041K0JQ<br>051V4KY<br>041L0AH<br>041L4KH<br>041L0KQ<br>03170KD<br>03140ZD<br>061V49Y<br>0312094<br>051G0ZY<br>041M4ZL<br>061T4JY<br>03150A2<br>03150K1

© 2023 Kim KM et al. *JAMA Network Open.*

				041K0JS<br>041K0AH<br>0315094<br>041K4ZP<br>031709F<br>0312097<br>03150J4<br>06130KY<br>041N0JS<br>041N4ZP<br>041K4JQ
Vascular	Vascular bypass and shunt, not heart	aorta-renal bypass	39.24	0410095<br>04100K4<br>04104A5<br>04104A4<br>04100Z4<br>0410495<br>04104Z4<br>04104K3<br>04104J3<br>04104A3<br>04104Z3<br>04104J4<br>04100Z5<br>04100A5<br>04100J3<br>04100A4<br>04100J5<br>04104K4<br>0410093<br>04100K5<br>04104J5<br>0410494<br>04104Z5<br>04100J4<br>04100Z3<br>04100K3<br>0410493<br>04104K5<br>04100A3<br>0410094
		intra-abdomin shunt nec	39.26	041J49D<br>041J099<br>041E4KP<br>041C49B<br>041E4JD<br>041D0JC<br>0410490<br>041F4KG<br>041C4K2<br>041C0Z5<br>041J09B<br>041H49P

		041F0J9
		041E0KG
		04140A3
		041C4K1
		041J4ZF
		04140Z4
		041C0Z8
		041E49B
		041F4K9
		041C4ZB
		041J0KQ
		041F49Q
		041H0ZP
		041D4A4
		041H49D
		041D0Z3
		041C0Z4
		041D0J0
		041E4ZP
		041E0ZP
		041D4A8
		041C0Z7
		041D0Z8
		041H49F
		041C0KC
		041C0ZQ
		041C4J2
		04104A2
		041C4A6
		041D0K1
		041E0KD
		041F0JB
		041J0AD
		041C4J4
		041F4KF
		041D09D
		041D4K5
		041E49C
		041C09C
		041E0AQ
		041E4JG
		041F4ZD
		041D4A6
		041C4JG
		041E09F
		041D0K9
		041J4KQ
		04140J3
		041F0KP
		041D0A4
		041C4A2
		04140K5
		041J0AG
		041D4K8

© 2023 Kim KM et al. *JAMA Network Open.*

		041D094
		041F0AG
		041C0Z3
		041D49R
		041F4ZF
		041D4Z0
		041F4KQ
		041H4JF
		041D0AD
		041H4KP
		041H4J9
		041E0KC
		041D4JC
		04100K0
		04144A3
		041E4JQ
		041F49C
		041D4A9
		041C0J3
		041F4KD
		041E0JF
		041C0KD
		041H4AG
		041C49C
		041F4JF
		041E4JP
		041C0A7
		041J0KB
		041F099
		041J09D
		041D4AB
		041C0Z9
		041D4Z8
		041D0Z6
		041D4K7
		041D4Z7
		041J49F
		041H4ZQ
		04104K2
		041H4KC
		041D0JB
		041F0ZB
		041C4Z6
		041C09R
		041C4A3
		041C0ZR
		041C0Z6
		041J4AD
		041D0AG
		041D4JB
		041E0AG
		041D09G
		041C0A8
		041D49D

© 2023 Kim KM et al. *JAMA Network Open.*

		041D4J5
		0414495
		041H4AP
		041C0K9
		041D0J8
		041C4KF
		041C09F
		041J0ZF
		041H0AQ
		041F0Z9
		041J0KG
		041C090
		041C0A3
		041C0KR
		041J09G
		041J4ZG
		041D4K2
		041E0KB
		041D0KF
		041F49P
		041C49Q
		041C4ZG
		041J0KD
		041H09G
		041C0Z2
		041H4AD
		041J0AC
		041C4AD
		0410090
		041D0ZF
		041H0ZC
		041H0A9
		041D4Z2
		041E4K9
		041C0J1
		041H0KF
		041J4ZQ
		041D0A9
		041F4KP
		04144K5
		041F49D
		041E4AD
		041C0J9
		041J4KP
		041E0AB
		041C4JC
		041D4ZQ
		041F09P
		041C497
		041D095
		041D0K7
		041F09Q
		041D4A0
		041D0ZD

© 2023 Kim KM et al. *JAMA Network Open.*

		041C4J3<br>041C4K0<br>041D0A5<br>041D0J2<br>041F0ZG<br>041E0ZD<br>041C4J7<br>041J4KC<br>041C4J6<br>041E4JB<br>041J49P<br>041C4A9<br>04100Z1<br>041D0J7<br>041J09Q<br>04100J0<br>04104Z2<br>041C097<br>041D0Z1<br>041H4K9<br>041C4Z9<br>041C0A6<br>041D0ZB<br>041D4AR<br>041C495<br>041D0J1<br>041C091<br>041F0KF<br>041C0A1<br>041C0K6<br>041D4KR<br>041D49Q<br>041E4JF<br>041D498<br>04140A5<br>041C4Z2<br>041D0A8<br>041J09F<br>041C4ZQ<br>04140J5<br>041D0Z2<br>041C0AF<br>041D0A2<br>041E0JD<br>041D0A7<br>041D499<br>04144J3<br>041C49R<br>041J4AB<br>041C0J7<br>041F09C<br>041E499<br>041D49F<br>041D4Z9

		04140Z3
		041C4JB
		041D4KC
		041C09D
		04100K2
		041C09G
		041C4JR
		041C496
		041F0KD
		04104K1
		041J0ZD
		041D0K3
		041F0AC
		041H0J9
		041H0KB
		041E09Q
		041H4ZC
		041D49G
		041H4JP
		041H4KF
		041C4Z3
		041C0A5
		041D4J8
		041D0K0
		041E49F
		041C4KD
		041D09C
		041J4ZD
		041D497
		04104J2
		041E0KF
		041C49F
		041C4K5
		041E4A9
		041C0KB
		04144A5
		041D4A3
		041E4AP
		041D4ZR
		041E0ZC
		041H099
		041D4A1
		04104J0
		041H4JQ
		041C0K8
		041F4AB
		041C499
		04140Z5
		041D0KC
		041D0AR
		041D0A0
		041E4ZG
		04100K1
		041C0AR

© 2023 Kim KM et al. *JAMA Network Open.*

		041C0ZG
		041E49G
		041C4Z1
		041D4J1
		041D0A3
		041E0JP
		041J0AP
		041C490
		041C4A4
		041F4JP
		041D4J6
		041D0Z7
		041E4AF
		04140A4
		041C4ZD
		041H4ZF
		041C0Z0
		041D4ZD
		041F0ZQ
		041H0JG
		041E0JC
		041E49P
		041D0JR
		041J0KF
		041D4JR
		041C0K2
		041D4K0
		04144K3
		041F09B
		041C4J5
		041D096
		041C094
		041H4Z9
		041J0ZC
		041C498
		041D0ZG
		041C492
		041C0J2
		04140J4
		041E4KB
		041F09F
		041D0JQ
		041E4KD
		041H4AF
		041J4AC
		041H499
		041F4AG
		041D4Z6
		041C4KG
		041F0KB
		041F0AD
		041C0ZC
		041C4AG
		041J09P

		041F4ZQ
		041F4JB
		041C4K7
		041D4K9
		041D4KF
		041F0JG
		041D493
		041E4KF
		041E09B
		041C0K5
		041J0AB
		041F0AB
		041H4JC
		041D0AF
		041D4JG
		041H0Z9
		041C0JG
		041J4Z9
		041E4AB
		0414493
		041C4K8
		041D0AC
		041C4A1
		041D4AC
		0414494
		041E4AC
		041D4AD
		041D0K2
		041D492
		041D0ZQ
		04144J4
		041C0J6
		041F0JP
		041H0AG
		041C4K3
		041E09C
		041J49B
		041J4JQ
		041C098
		041H4ZB
		041E0A9
		041C0AQ
		041H09C
		041F0ZD
		041D4KQ
		041J4KG
		041J49Q
		041D4J9
		041J4KF
		041H4ZD
		041J4ZC
		041D4AQ
		041H0ZD
		04144J5

		041H4KG
		041D4A7
		041F0JF
		041E0Z9
		04144A4
		041D0K4
		041D0Z5
		04144K4
		041D494
		041E0ZG
		041C0J5
		041C0K7
		041E4J9
		041J49C
		041C4KR
		041J09C
		041C4AR
		041J0A9
		041C0J8
		041C0JF
		041C4J8
		041H0ZG
		041D0A6
		041C49D
		041H4JB
		0414093
		041F499
		041F4JG
		041H0AB
		041D0J5
		041C4JD
		041E4ZF
		041C099
		041D09R
		0414094
		041E4JC
		041H09F
		041H09B
		041C4J0
		041D0Z0
		041E4ZD
		041C0A4
		041F4ZP
		041C0JR
		041C4JQ
		041E4AQ
		041H4JG
		041C4K4
		041D491
		041D4K6
		041C0AB
		041D4J7
		041D4K3
		041C093

© 2023 Kim KM et al. *JAMA Network Open.*

		04140K3<br>04104A1<br>041C0J0<br>041D4ZB<br>041D09F<br>04144Z3<br>041D0ZC<br>041D4ZF<br>041E0ZQ<br>041C0K1<br>041J0ZQ<br>041J4JF<br>041H09P<br>041J4J9<br>041C09B<br>041J4ZP<br>041D4Z1<br>041D09Q<br>041D4ZC<br>041E4KC<br>041H0KC<br>041D4AF<br>041J4AG<br>041E0JB<br>041F4ZC<br>041C4J9<br>041C493<br>041D0Z4<br>041D090<br>04140K4<br>041H49G<br>041C4Z7<br>041D0ZR<br>041C0JC<br>041D092<br>041D0JG<br>041J49G<br>041J4KD<br>041C494<br>04104J1<br>041F0K9<br>041J4A9<br>041C4Z4<br>04144Z4<br>041C0K4<br>041H0AF<br>041D4A2<br>041E0ZF<br>041C4J1<br>04100Z0<br>041C4A7<br>041C096<br>041D4Z5<br>04100Z2

© 2023 Kim KM et al. *JAMA Network Open.*

		041J4JB
		041D091
		041H09Q
		041H0JP
		041D4AG
		041J4JP
		041E0JQ
		041C0ZD
		041H0K9
		041C0KQ
		041E49Q
		041D0J3
		041D0K6
		041J4K9
		041F0AF
		041J0J9
		041C4A5
		041J0ZP
		041F4AQ
		041F0KQ
		041C49G
		041H49B
		041F4J9
		041E0K9
		041F4A9
		041C0JQ
		04104K0
		041E49D
		04104A0
		041H0AP
		041H0JB
		041D0AB
		041H0KQ
		041F4AD
		041J4JD
		041J0K9
		0410091
		041D0KQ
		041H0JF
		041D4J3
		041D0J4
		04104Z1
		041H4AB
		041D49C
		041D4J0
		041E4KQ
		041E0J9
		041J4KB
		041J0KP
		041J4AQ
		041C0ZF
		041D0JD
		041D0J6
		041C4K9

© 2023 Kim KM et al. *JAMA Network Open.*

		041D09B
		041C0K0
		041C095
		041J4JG
		041J0KC
		04100J1
		041C4AF
		041C0Z1
		041F4JQ
		041H49Q
		041D4Z4
		041C4AC
		041H4JD
		041C491
		0410092
		041C0AG
		041H0ZF
		041H0AD
		041C4ZF
		041E0KQ
		041J0ZG
		0410492
		041D0A1
		041F0ZF
		041H0JD
		041F4KC
		041C4Z8
		041E4KG
		041C0K3
		041E4AG
		041D4K1
		041F4JD
		041C4Z5
		041D4ZG
		041D4J4
		041F0KC
		041F4Z9
		041E09P
		041F4KB
		041F0JQ
		041D0Z9
		041C0ZB
		04104Z0
		041F49G
		041D0KR
		041H4ZG
		041C0JD
		041C4AQ
		041E4ZQ
		041C4JF
		041F0KG
		04100A2
		041D0K8
		041D097

© 2023 Kim KM et al. *JAMA Network Open.*

		041D4JD
		041E4Z9
		041D4KB
		041D098
		041H4KQ
		041J0JF
		041C0AD
		041H49C
		041H0JC
		041F0JC
		041D0J9
		041C4AB
		041C4A8
		041H0AC
		041H4KB
		041D496
		04100A1
		041C0A0
		041J499
		041F49F
		041F4ZG
		041C4Z0
		041F4ZB
		041J0JP
		041J4ZB
		041J0Z9
		041F4JC
		041F09D
		041C0KF
		041D093
		041E0JG
		041C4KB
		041D490
		041C092
		041D0K5
		041C4ZC
		041E09G
		041C4K6
		041D4JF
		041D099
		041D49B
		041F0ZP
		0414095
		041H4ZP
		041D0KD
		041J0JQ
		041F0ZC
		041D0JF
		041F4AF
		041D495
		04144Z5
		041D4KG
		041C0A2
		041E0AF

		041C0AC<br>041H0ZQ<br>041H4AC<br>041D4A5<br>0410491<br>041J0JC<br>041C0KG<br>041D0KG<br>041H0ZB<br>041E0AD<br>041F49B<br>041E4ZB<br>041D4KD<br>041D4JQ<br>041H0KP<br>041F4AC<br>041C4KQ<br>041J0AF<br>041D4K4<br>041E0KP<br>041H0KG<br>041J0JG<br>041J0ZB<br>041F4AP<br>041H09D<br>041D4Z3<br>041H0KD<br>041D0KB<br>041E09D<br>041C0A9<br>041F09G<br>041F0A9<br>041F0JD<br>041J4AP<br>041E4ZC<br>041J0AQ<br>041C4KC<br>04100A0<br>041J4JC<br>041D4J2<br>041C09Q<br>041E099<br>041E0AC<br>041C4ZR<br>041J0JD<br>041E0ZB<br>041C0J4<br>041C4A0<br>041H0JQ<br>041F0AP<br>041H4AQ<br>041E0AP<br>041J0JB<br>04100J2

© 2023 Kim KM et al. *JAMA Network Open.*

Neuro		extracran-intracr bypass	39.28	041C0JB<br>041J4AF<br>041H4A9<br>041F0AQ<br>041H4KD<br>041D0AQ<br>031H0JJ<br>031N0AK<br>031H09J<br>031M0ZJ<br>031H0AJ<br>031J0AK<br>031K0ZJ<br>031S0ZG<br>031J0ZK<br>031H0KJ<br>031M0AJ<br>031L0JK<br>031S09G<br>031K0AJ<br>031S0AG<br>031T0AG<br>031N09K<br>031K0KJ<br>031H0ZJ<br>031T09G<br>031N0ZK<br>031M0KJ<br>031T0KG<br>031S0KG<br>031L0ZK<br>031J0KK<br>031L09K<br>031J0JK<br>031N0KK<br>031S0JG<br>031M0JJ<br>031T0ZG<br>031K0JJ<br>031L0AK<br>031T0JG<br>031M09J<br>031N0JK<br>031L0KK<br>031K09J<br>031J09K
Neuro	Neuro Vascular	intracran vessel incis	38.01	05CL4ZZ<br>05CL0ZZ<br>03CG0ZZ
		occlus intracran ves nec	38.81	03LG3ZZ<br>05LL0CZ<br>05LL4ZZ<br>05LL3CZ<br>05LL4DZ

© 2023 Kim KM et al. *JAMA Network Open.*

				05LL4CZ<br>05LL3DZ<br>05LL0ZZ<br>03LG0ZZ<br>03LG0CZ<br>03LG4ZZ<br>05LL3ZZ<br>05LL0DZ<br>03LG4CZ<br>03LG3CZ
Vascular	Embolectomy and endarterectomy of lower limbs	lower limb endarterect	38.18	04CR0ZZ<br>04CY0ZZ<br>04CY3ZZ<br>04CS4ZZ<br>04CV0ZZ<br>04CP3ZZ<br>04CU0ZZ<br>04CW4ZZ<br>04CN4ZZ<br>04CR4ZZ<br>04CS0ZZ<br>04CK3ZZ<br>04CU4ZZ<br>04CK0ZZ<br>04CW3ZZ<br>04CP0ZZ<br>04CT4ZZ<br>04CT0ZZ<br>04CU3ZZ<br>04CS3ZZ<br>04CW0ZZ<br>04CK4ZZ<br>04CV4ZZ<br>04CV3ZZ<br>04CN0ZZ<br>04CT3ZZ<br>04CR3ZZ<br>04CQ4ZZ<br>04CQ3ZZ<br>04CP4ZZ<br>04CY4ZZ<br>04CM0ZZ<br>04CQ0ZZ<br>04CL3ZZ<br>04CL4ZZ<br>04CL0ZZ<br>04CM3ZZ<br>04CN3ZZ<br>04CM4ZZ
Vascular	Emboletomy of Central Vessels	endarterectomy of aorta	38.14	02CW0ZZ<br>04C03ZZ<br>02CW4ZZ

© 2023 Kim KM et al. *JAMA Network Open.*

				04C04ZZ
				04C00ZZ
				02CW3ZZ
		thoracic endarterectomy	38.15	03C04ZZ
				03C14ZZ
				02CQ0ZZ
				03C40ZZ
				02CQ4ZZ
				02CT4ZZ
				03C33ZZ
				03C34ZZ
				02CP3ZZ
				02CV3ZZ
				02CV4ZZ
				03C30ZZ
				02CR4ZZ
				03C44ZZ
				02CR3ZZ
				03C03ZZ
				02CS3ZZ
				02CS4ZZ
				03C10ZZ
				03C00ZZ
				03C43ZZ
				02CP0ZZ
				03C24ZZ
				02CT0ZZ
				02CS0ZZ
				03C20ZZ
				02CR0ZZ
				02CQ3ZZ
				03C23ZZ
				02CP4ZZ
				02CV0ZZ
				03C13ZZ
				02CT3ZZ
		abdominal endarterectomy	38.16	04CF0ZZ
				04C44ZZ
				04C60ZZ
				04CH4ZZ
				04C43ZZ
				04CC4ZZ
				04CF3ZZ
				04C10ZZ
				04CA4ZZ
				04CA3ZZ
				04CE3ZZ
				04CJ3ZZ
				04C80ZZ
				04C24ZZ
				04CB3ZZ
				04CC0ZZ
				04C14ZZ
				04C84ZZ

© 2023 Kim KM et al. *JAMA Network Open.*

				04C83ZZ<br>04CD4ZZ<br>04C53ZZ<br>04CD3ZZ<br>04C54ZZ<br>04C34ZZ<br>04CF4ZZ<br>04CE4ZZ<br>04C74ZZ<br>04C30ZZ<br>04CB0ZZ<br>04CJ4ZZ<br>04C20ZZ<br>04C33ZZ<br>04CH3ZZ<br>04C94ZZ<br>04C50ZZ<br>04CA0ZZ<br>04C64ZZ<br>04C63ZZ<br>04CJ0ZZ<br>04C73ZZ<br>04C70ZZ<br>04CC3ZZ<br>04C40ZZ<br>04C13ZZ<br>04C90ZZ<br>04CH0ZZ<br>04C23ZZ<br>04CB4ZZ<br>04CE0ZZ<br>04C93ZZ<br>04CD0ZZ
Vascular	Other Major Vasc Repair/Intervention	incision of aorta	38.04	04C04ZZ<br>02CW0ZZ<br>04C00ZZ<br>02CW3ZZ<br>02CW4ZZ<br>04C03ZZ
		thoracic vessel inc nec	38.05	03C30ZZ<br>02CT3ZZ<br>03C44ZZ<br>03C43ZZ<br>05C03ZZ<br>05C43ZZ<br>02HS4DZ<br>05C40ZZ<br>02HS42Z<br>05C34ZZ<br>05C30ZZ<br>05C64ZZ<br>02HT3DZ<br>03C23ZZ

© 2023 Kim KM et al. *JAMA Network Open.*

		03C20ZZ<br>05C00ZZ<br>02CQ3ZZ<br>02CP0ZZ<br>03C04ZZ<br>03C00ZZ<br>02HR4DZ<br>02CR3ZZ<br>02HQ0DZ<br>02CV0ZZ<br>02HT0DZ<br>02CP3ZZ<br>03C33ZZ<br>02HT42Z<br>02HW4DZ<br>02HS32Z<br>02CS3ZZ<br>02HS0DZ<br>02CQ0ZZ<br>03C03ZZ<br>02HW02Z<br>02HP3DZ<br>02CT4ZZ<br>05C54ZZ<br>05C04ZZ<br>03C14ZZ<br>02HP4DZ<br>02CV4ZZ<br>02HS3DZ<br>02HW42Z<br>02CR0ZZ<br>02HQ3DZ<br>05C14ZZ<br>03C13ZZ<br>02HS02Z<br>05C44ZZ<br>03C40ZZ<br>02HW3DZ<br>02HP0DZ<br>03C24ZZ<br>05C13ZZ<br>03C10ZZ<br>02HQ4DZ<br>02CV3ZZ<br>02HR0DZ<br>02HT4DZ<br>02HW0DZ<br>05C60ZZ<br>03C34ZZ<br>05C33ZZ<br>02CR4ZZ<br>02CP4ZZ<br>02CT0ZZ<br>02HT02Z

© 2023 Kim KM et al. *JAMA Network Open.*

				02HR3DZ<br>05C50ZZ<br>05C10ZZ<br>02CQ4ZZ<br>05C53ZZ<br>02HT32Z<br>05C63ZZ<br>02CS0ZZ<br>02HW32Z<br>02CS4ZZ
		abdomen artery incision	38.06	04C54ZZ<br>04C13ZZ<br>04CE0ZZ<br>04C10ZZ<br>04CH0ZZ<br>04C30ZZ<br>04CH4ZZ<br>04CJ3ZZ<br>04CE4ZZ<br>04C84ZZ<br>04CJ0ZZ<br>04C53ZZ<br>04C24ZZ<br>04C63ZZ<br>04C83ZZ<br>04CA3ZZ<br>04CC4ZZ<br>04C50ZZ<br>04C74ZZ<br>04C44ZZ<br>04C34ZZ<br>04C60ZZ<br>04CF3ZZ<br>04C43ZZ<br>04C64ZZ<br>04C20ZZ<br>04CD4ZZ<br>04CF0ZZ<br>04CC0ZZ<br>04C14ZZ<br>04CD3ZZ<br>04C40ZZ<br>04CH3ZZ<br>04CJ4ZZ<br>04C73ZZ<br>04CB0ZZ<br>04C94ZZ<br>04CE3ZZ<br>04C93ZZ<br>04C33ZZ<br>04CA0ZZ<br>04C70ZZ<br>04CA4ZZ<br>04CB3ZZ

© 2023 Kim KM et al. *JAMA Network Open.*

				04CC3ZZ<br>04C90ZZ<br>04C80ZZ<br>04C23ZZ<br>04CB4ZZ<br>04CF4ZZ<br>04CD0ZZ
		abdominal vein incision	38.07	06C04ZZ<br>06C54ZZ<br>06C70ZZ<br>06C03ZZ<br>06C43ZZ<br>06CH3ZZ<br>06CJ0ZZ<br>06CH0ZZ<br>06C84ZZ<br>06C60ZZ<br>06C83ZZ<br>06C63ZZ<br>06CB3ZZ<br>06CJ3ZZ<br>06C10ZZ<br>06C13ZZ<br>06CF3ZZ<br>06C40ZZ<br>06C50ZZ<br>06CG0ZZ<br>06CD4ZZ<br>06C73ZZ<br>06C80ZZ<br>06CC3ZZ<br>06C94ZZ<br>06CG3ZZ<br>06CD3ZZ<br>06CH4ZZ<br>06C53ZZ<br>06C00ZZ<br>06CF4ZZ<br>06C64ZZ<br>06CD0ZZ<br>06CJ4ZZ<br>06C44ZZ<br>06CB4ZZ<br>06CC0ZZ<br>06C93ZZ<br>06CF0ZZ<br>06C24ZZ<br>06CG4ZZ<br>06C90ZZ<br>06C74ZZ<br>06C23ZZ<br>06CB0ZZ<br>06C14ZZ<br>06C20ZZ

				06CC4ZZ
		thor vessel resect/anast	38.35	05B60ZZ
				03B34ZZ
				05B04ZZ
				02BP0ZZ
				03B24ZZ
				03B14ZZ
				02BV0ZZ
				02BS0ZZ
				03B44ZZ
				03B30ZZ
				05B40ZZ
				02BT4ZZ
				05B30ZZ
				03B00ZZ
				05B10ZZ
				02BS4ZZ
				05B00ZZ
				03B40ZZ
				05B14ZZ
				02BT0ZZ
				03B10ZZ
				05B64ZZ
				02BQ4ZZ
				05B34ZZ
				03B04ZZ
				03B20ZZ
				05B44ZZ
				02BR4ZZ
				02BP4ZZ
				05B54ZZ
				02BV4ZZ
				02BQ0ZZ
				02BR0ZZ
				05B50ZZ
		abd vessel resect/anast	38.36	04B74ZZ
				04B60ZZ
				04BE0ZZ
				04BC0ZZ
				04BH0ZZ
				04B14ZZ
				04B80ZZ
				04B30ZZ
				04BJ4ZZ
				04BA0ZZ
				04BD0ZZ
				04BF0ZZ
				04B40ZZ
				04BB4ZZ
				04BF4ZZ
				04BE4ZZ
				04BC4ZZ
				04B50ZZ
				04B34ZZ

© 2023 Kim KM et al. *JAMA Network Open.*

				04B94ZZ
				04B54ZZ
				04BA4ZZ
				04B24ZZ
				04B44ZZ
				04B10ZZ
				04B90ZZ
				04BJ0ZZ
				04BD4ZZ
				04B64ZZ
				04B20ZZ
				04B84ZZ
				04BB0ZZ
				04BH4ZZ
				04B70ZZ
		resect thorac ves w repl	38.45	05R10KZ
				02RV4JZ
				05R40KZ
				05R54JZ
				05R607Z
				02RT48Z
				02RQ0KZ
				03R20KZ
				02RV0KZ
				02RS4KZ
				02RQ08Z
				02RR0JZ
				05R10JZ
				05R007Z
				03R00KZ
				05R14JZ
				05R40JZ
				03R00JZ
				03R10JZ
				02RR4KZ
				05R34JZ
				05R60JZ
				02RP08Z
				03R44KZ
				02RV07Z
				05R30JZ
				02RR07Z
				02RQ4JZ
				03R20JZ
				05R347Z
				05R407Z
				02RV48Z
				05R547Z
				02RT0JZ
				02RQ0JZ
				02RQ48Z
				02RV0JZ
				03R24JZ
				03R24KZ

© 2023 Kim KM et al. *JAMA Network Open.*

		02RW4KZ
		03R40JZ
		02RT4KZ
		05R507Z
		05R60KZ
		03R207Z
		02RR0KZ
		02RQ4KZ
		02RS0KZ
		03R34KZ
		02RS0JZ
		03R04JZ
		05R64JZ
		05R04KZ
		05R30KZ
		02RR4JZ
		05R00KZ
		02RS47Z
		02RR08Z
		02RP0KZ
		05R44JZ
		05R64KZ
		05R50KZ
		02RT08Z
		02RT07Z
		03R447Z
		05R44KZ
		05R14KZ
		03R247Z
		03R347Z
		05R54KZ
		02RP07Z
		05R50JZ
		03R14JZ
		03R30JZ
		03R30KZ
		02RW4JZ
		05R307Z
		02RW07Z
		02RS08Z
		02RW0KZ
		02RR47Z
		02RS4JZ
		02RV47Z
		03R04KZ
		02RT47Z
		03R007Z
		05R34KZ
		02RV08Z
		05R147Z
		02RP4KZ
		02RT4JZ
		05R04JZ
		02RT0KZ

				02RS48Z
				02RW0JZ
				02RP0JZ
				02RP48Z
				02RW08Z
				03R107Z
				05R447Z
				05R047Z
				02RV4KZ
				05R00JZ
				03R047Z
				02RQ07Z
				03R307Z
				03R44JZ
				03R10KZ
				02RP47Z
				02RQ47Z
				02RW47Z
				02RR48Z
				02RW48Z
				02RP4JZ
				05R107Z
				05R647Z
				03R147Z
				03R34JZ
				03R14KZ
				03R407Z
				02RS07Z
				03R40KZ
		abd artery resec w repla	38.46	04RH07Z
				04RB47Z
				04RC4KZ
				04R747Z
				04R90JZ
				04RC4JZ
				04RH4KZ
				04R14JZ
				04RJ47Z
				04R64KZ
				04RJ0KZ
				04R70JZ
				04R407Z
				04R74JZ
				04RE0JZ
				04R847Z
				04R20JZ
				04R34JZ
				04RF0JZ
				04RF0KZ
				04RD0KZ
				04R207Z
				04R44KZ
				04RB0JZ
				04RA0KZ

© 2023 Kim KM et al. *JAMA Network Open.*

		04RE4JZ<br>04R10JZ<br>04RA07Z<br>04R107Z<br>04RA47Z<br>04R147Z<br>04R40KZ<br>04R54KZ<br>04R74KZ<br>04RJ0JZ<br>04R90KZ<br>04RC47Z<br>04R607Z<br>04RE07Z<br>04RE4KZ<br>04RH0KZ<br>04RH0JZ<br>04RC07Z<br>04RH4JZ<br>04RE0KZ<br>04R50JZ<br>04RE47Z<br>04RF47Z<br>04R94KZ<br>04R54JZ<br>04R947Z<br>04R707Z<br>04R60KZ<br>04RJ4KZ<br>04RD47Z<br>04R60JZ<br>04R247Z<br>04R14KZ<br>04R30JZ<br>04RF07Z<br>04R447Z<br>04RD07Z<br>04RA0JZ<br>04RH47Z<br>04R40JZ<br>04R20KZ<br>04RD4JZ<br>04R94JZ<br>04RA4KZ<br>04R50KZ<br>04RB0KZ<br>04R507Z<br>04R24KZ<br>04R347Z<br>04R84JZ<br>04R84KZ<br>04RC0KZ<br>04RD0JZ<br>04R547Z

© 2023 Kim KM et al. *JAMA Network Open.*

				04R64JZ
				04R307Z
				04RB4KZ
				04RC0JZ
				04RB4JZ
				04R70KZ
				04R80KZ
				04R647Z
				04R80JZ
				04R807Z
				04RD4KZ
				04R44JZ
				04RJ07Z
				04R907Z
				04RB07Z
				04RJ4JZ
				04R30KZ
				04R10KZ
				04RA4JZ
				04R24JZ
				04RF4KZ
				04RF4JZ
				04R34KZ
		thoracic vessel excision	38.65	05503ZZ
				03B04ZZ
				05560ZZ
				05543ZZ
				05544ZZ
				05553ZZ
				02BP4ZZ
				03504ZZ
				05B64ZZ
				03B13ZZ
				02BR4ZZ
				05B13ZZ
				05B14ZZ
				025T0ZZ
				05510ZZ
				02BT0ZZ
				05B54ZZ
				02BV0ZZ
				03500ZZ
				025T3ZZ
				02BS3ZZ
				025V4ZZ
				05B43ZZ
				025S0ZZ
				025W0ZZ
				03534ZZ
				03523ZZ
				05554ZZ
				03B23ZZ
				05533ZZ
				03B30ZZ

© 2023 Kim KM et al. *JAMA Network Open.*

		025P3ZZ<br>05530ZZ<br>02BS4ZZ<br>05B30ZZ<br>025Q0ZZ<br>05550ZZ<br>02BS0ZZ<br>025V0ZZ<br>05564ZZ<br>02BV4ZZ<br>025P0ZZ<br>05B04ZZ<br>03B20ZZ<br>025T4ZZ<br>02BV3ZZ<br>025Q4ZZ<br>03503ZZ<br>03544ZZ<br>03510ZZ<br>025S4ZZ<br>025R0ZZ<br>05B63ZZ<br>03B33ZZ<br>05B10ZZ<br>03530ZZ<br>03540ZZ<br>05540ZZ<br>025W3ZZ<br>05B03ZZ<br>05B53ZZ<br>03B14ZZ<br>03B34ZZ<br>03514ZZ<br>05B44ZZ<br>02BQ4ZZ<br>03B43ZZ<br>03520ZZ<br>02BT4ZZ<br>025V3ZZ<br>025R4ZZ<br>03B44ZZ<br>03524ZZ<br>02BQ3ZZ<br>05B33ZZ<br>02BR0ZZ<br>025P4ZZ<br>025Q3ZZ<br>05500ZZ<br>03B40ZZ<br>03B10ZZ<br>02BR3ZZ<br>05B50ZZ<br>03B00ZZ<br>05B34ZZ

© 2023 Kim KM et al. *JAMA Network Open.*

				03513ZZ
				025R3ZZ
				05B60ZZ
				025W4ZZ
				05504ZZ
				05534ZZ
				02BP0ZZ
				05563ZZ
				05B00ZZ
				03543ZZ
				025S3ZZ
				05513ZZ
				02BQ0ZZ
				05514ZZ
				02BT3ZZ
				03B24ZZ
				05B40ZZ
				03533ZZ
				02BP3ZZ
				02BW3ZZ
				03B03ZZ
		vasc proc revision nec	39.49	05CY0ZZ
				06CY3ZZ
				04QY0ZZ
				03CY3ZZ
				06QY0ZZ
				03CY0ZZ
				05CY4ZZ
				05QY3ZZ
				05QY4ZZ
				05CY3ZZ
				04QY4ZZ
				04QY3ZZ
				06CY4ZZ
				03QY4ZZ
				04CY0ZZ
				06CY0ZZ
				03QY0ZZ
				06QY3ZZ
				03QY3ZZ
				05QY0ZZ
				03CY4ZZ
				04CY4ZZ
				04CY3ZZ
				06QY4ZZ
		aneurysm repair nec	39.52	02VW0ZZ
				06VS4ZZ
				04VM4ZZ
				06VG0ZZ
				03V64ZZ
				03VQ0ZZ
				06VH0ZZ
				05VG4ZZ
				06VQ0ZZ

© 2023 Kim KM et al. *JAMA Network Open.*

		05VL4ZZ<br>06V50ZZ<br>03V20ZZ<br>05V10ZZ<br>06VC4ZZ<br>03VR0ZZ<br>02VS0ZZ<br>05VA0ZZ<br>04VK0ZZ<br>05V70ZZ<br>03V54ZZ<br>04V20ZZ<br>03V14ZZ<br>05V14ZZ<br>04VY4ZZ<br>05VS4ZZ<br>04V34ZZ<br>06V54ZZ<br>06VG4ZZ<br>06VR4ZZ<br>04VJ0ZZ<br>06VP0ZZ<br>03VN0ZZ<br>03VF0ZZ<br>05V60ZZ<br>05VV0ZZ<br>06V30ZZ<br>02VT0DZ<br>06VQ4ZZ<br>06VD4ZZ<br>03V24ZZ<br>03VL0ZZ<br>05VB4ZZ<br>04VD4ZZ<br>03VU4ZZ<br>05V90ZZ<br>05VY4ZZ<br>06V70ZZ<br>05VP4ZZ<br>03VC0ZZ<br>05VA4ZZ<br>04VN4ZZ<br>04V24ZZ<br>04VF4ZZ<br>05VM0ZZ<br>03V40ZZ<br>03V04ZZ<br>04V50ZZ<br>04V80ZZ<br>04VS0ZZ<br>06V64ZZ<br>06V44ZZ<br>04V00DZ<br>04VQ4ZZ

		04VS4ZZ
		06V60ZZ
		05VD4ZZ
		03V30ZZ
		04VD0ZZ
		06VY0ZZ
		05VN4ZZ
		04V54ZZ
		03VY0ZZ
		04VU0ZZ
		03V70ZZ
		03VM0ZZ
		05VH4ZZ
		06V24ZZ
		04VT0ZZ
		03VY4ZZ
		05VM4ZZ
		02VP0ZZ
		06VM0ZZ
		03V44ZZ
		06VN4ZZ
		04VU4ZZ
		04VE4ZZ
		04VJ4ZZ
		05V44ZZ
		04V04ZZ
		05V50ZZ
		06VY0DZ
		06V20ZZ
		04VK4ZZ
		03VR4ZZ
		04VL4ZZ
		03V80ZZ
		06VY4ZZ
		06VR0ZZ
		04VW4ZZ
		04VY0ZZ
		03VD4ZZ
		03VJ0ZZ
		02VT4ZZ
		04V64ZZ
		06V84ZZ
		05VC0ZZ
		05VT0ZZ
		04VV4ZZ
		04V44ZZ
		05VL0ZZ
		03VB4ZZ
		03VA0ZZ
		02VT4DZ
		05VY0DZ
		02VQ4ZZ
		06V94ZZ
		03VP4ZZ

© 2023 Kim KM et al. *JAMA Network Open.*

		03V34ZZ<br>03V00ZZ<br>06V14ZZ<br>02VW4DZ<br>04VB0ZZ<br>04VB4ZZ<br>04VC0ZZ<br>04VV0ZZ<br>05VR0ZZ<br>05VQ4ZZ<br>04V60ZZ<br>06V40ZZ<br>05V34ZZ<br>06VF0ZZ<br>02VS4ZZ<br>02VS4DZ<br>02VS0DZ<br>06VB4ZZ<br>03VC4ZZ<br>06VD0ZZ<br>03V90ZZ<br>06VB0ZZ<br>03VQ4ZZ<br>04V90ZZ<br>06V34ZZ<br>06VJ4ZZ<br>06VV0ZZ<br>05V00ZZ<br>05V64ZZ<br>04VL0ZZ<br>05VS0ZZ<br>03V10ZZ<br>04VE0ZZ<br>03V50ZZ<br>05VT4ZZ<br>03VS4ZZ<br>04VH4ZZ<br>04VP0ZZ<br>02VR0DZ<br>03VU0ZZ<br>06VM4ZZ<br>03VG0ZZ<br>05VP0ZZ<br>05VR4ZZ<br>03VF4ZZ<br>05VB0ZZ<br>03VT4ZZ<br>03VS0ZZ<br>04VC4ZZ<br>06V90ZZ<br>03VD0ZZ<br>05VV4ZZ<br>02VR4ZZ<br>04VA4ZZ

© 2023 Kim KM et al. *JAMA Network Open.*

		03V60ZZ
		05V80ZZ
		05V40ZZ
		04V14ZZ
		04VR4ZZ
		03VN4ZZ
		02VR4DZ
		04VR0ZZ
		06VT0ZZ
		06V74ZZ
		05VC4ZZ
		06VS0ZZ
		06VN0ZZ
		02VQ4DZ
		06VV4ZZ
		02VP4DZ
		03VV4ZZ
		02VR4DT
		06VP4ZZ
		04VN0ZZ
		06VT4ZZ
		04VT4ZZ
		05VN0ZZ
		03VV0ZZ
		04V10ZZ
		02VR0ZT
		04V70ZZ
		04V00ZZ
		06VY4DZ
		04VA0ZZ
		05V74ZZ
		04VM0ZZ
		02VW4ZZ
		04V74ZZ
		04VF0ZZ
		06VJ0ZZ
		03V74ZZ
		06VF4ZZ
		03VT0ZZ
		03V84ZZ
		04V94ZZ
		05VQ0ZZ
		04V40ZZ
		06V80ZZ
		04VQ0ZZ
		02VR0ZZ
		03VK0ZZ
		03VB0ZZ
		03VJ4ZZ
		05VD0ZZ
		03VG4ZZ
		05VF4ZZ
		05VY4DZ
		04VH0ZZ

				05VH0ZZ
				05VG0ZZ
				05V30ZZ
				03VP0ZZ
				03VL4ZZ
				05VF0ZZ
				06VC0ZZ
				05V94ZZ
				05V84ZZ
				03VM4ZZ
				03V94ZZ
				02VQ0DZ
				02VP0DZ
				03VK4ZZ
				02VQ0ZZ
				02VR4ZT
				05V04ZZ
				05V54ZZ
				06VH4ZZ
				02VR0DT
				02VP4ZZ
				04VP4ZZ
				06V10ZZ
				04VW0ZZ
				03VA4ZZ
				02VT0ZZ
				03VH0ZZ
				03VH4ZZ
				05VY0ZZ
				04V84ZZ
				04V30ZZ
		rep vess w synth patch	39.57	03UU4JZ
				03UH0JZ
				03U93JZ
				03U70JZ
				03UC4JZ
				03U64JZ
				04U70JZ
				05U53JZ
				06UT4JZ
				04UJ3JZ
				04UA4JZ
				06UP4JZ
				04UW0JZ
				02UQ4JZ
				03U24JZ
				03UF4JZ
				06UT3JZ
				05U63JZ
				03U00JZ
				06UD3JZ
				05U14JZ
				05UQ4JZ
				06U74JZ

© 2023 Kim KM et al. *JAMA Network Open.*

		03UB3JZ<br>04UY4JZ<br>04UH3JZ<br>06UV4JZ<br>06U90JZ<br>03UU3JZ<br>02UV0JZ<br>05UH0JZ<br>06U14JZ<br>04U74JZ<br>06UR4JZ<br>06US4JZ<br>04UJ4JZ<br>04U94JZ<br>04U00JZ<br>06UQ3JZ<br>06UV0JZ<br>06UN4JZ<br>06U04JZ<br>06UF4JZ<br>05UC4JZ<br>03U23JZ<br>03U20JZ<br>06UH4JZ<br>05UA4JZ<br>06UG0JZ<br>06UV3JZ<br>03UB4JZ<br>03UP0JZ<br>03UM0JZ<br>04UP0JZ<br>05U70JZ<br>04UM4JZ<br>04UK4JZ<br>03UL0JZ<br>04UF0JZ<br>06UY0JZ<br>03UR4JZ<br>04U63JZ<br>05US0JZ<br>05U60JZ<br>05UP4JZ<br>05UF4JZ<br>05U44JZ<br>03U60JZ<br>06UT0JZ<br>04UA0JZ<br>06U93JZ<br>03U43JZ<br>04UT3JZ<br>03U90JZ<br>06UN3JZ<br>03UY0JZ<br>04UU4JZ

		04U64JZ<br>04U53JZ<br>06U54JZ<br>05U94JZ<br>05U54JZ<br>03U80JZ<br>03U84JZ<br>05U34JZ<br>04U34JZ<br>02US0JZ<br>05U84JZ<br>02UV3JZ<br>04UH4JZ<br>04U03JZ<br>03UH3JZ<br>05UY3JZ<br>05UF3JZ<br>03UT0JZ<br>06U73JZ<br>04U43JZ<br>03U40JZ<br>04UB3JZ<br>02UP3JZ<br>03UJ3JZ<br>06U13JZ<br>06UM3JZ<br>06U20JZ<br>03UC3JZ<br>06U80JZ<br>05UQ3JZ<br>06UG3JZ<br>03UC0JZ<br>05UL4JZ<br>04U13JZ<br>03UD3JZ<br>05UY0JZ<br>05UA0JZ<br>03UY3JZ<br>06U24JZ<br>04U14JZ<br>03UN0JZ<br>05U04JZ<br>03UJ4JZ<br>04UW4JZ<br>03U74JZ<br>06U84JZ<br>03UG0JZ<br>06UQ4JZ<br>03U54JZ<br>02UQ3JZ<br>03U03JZ<br>06U83JZ<br>04U24JZ<br>05UM4JZ

© 2023 Kim KM et al. *JAMA Network Open.*

		03UR0JZ<br>02US4JZ<br>03U33JZ<br>02UT4JZ<br>03UY4JZ<br>04UP4JZ<br>06UQ0JZ<br>04UL4JZ<br>05UB3JZ<br>03UH4JZ<br>05UD3JZ<br>05U10JZ<br>04UT0JZ<br>04UM0JZ<br>06UD0JZ<br>02UP0JZ<br>03UF0JZ<br>06UJ4JZ<br>05UQ0JZ<br>05UN3JZ<br>05UH4JZ<br>04UE0JZ<br>05U80JZ<br>06UM0JZ<br>02UT0JZ<br>04UR0JZ<br>03UA0JZ<br>05UV4JZ<br>06UD4JZ<br>04UU0JZ<br>05U00JZ<br>03UM4JZ<br>06U34JZ<br>04UU3JZ<br>04U40JZ<br>05UN0JZ<br>03UB0JZ<br>04UF4JZ<br>06UF3JZ<br>06U30JZ<br>04U83JZ<br>05UT4JZ<br>05U74JZ<br>06UC3JZ<br>04UK3JZ<br>03UP4JZ<br>04UN3JZ<br>02UV4JZ<br>04UF3JZ<br>06UN0JZ<br>04U20JZ<br>03UQ3JZ<br>05US4JZ<br>05UM3JZ

© 2023 Kim KM et al. *JAMA Network Open.*

		03U44JZ
		04UH0JZ
		04UQ0JZ
		03UG4JZ
		04US0JZ
		04UN0JZ
		06UH0JZ
		04UY3JZ
		06U94JZ
		06U23JZ
		05UD4JZ
		05UG0JZ
		06UB3JZ
		03UR3JZ
		03US4JZ
		06U44JZ
		06U10JZ
		04UL0JZ
		04U50JZ
		03UT3JZ
		06U40JZ
		06U43JZ
		03UL4JZ
		06U60JZ
		05UR0JZ
		04UD0JZ
		06UR3JZ
		03UN4JZ
		04U10JZ
		04UR4JZ
		05U43JZ
		06UJ3JZ
		06U50JZ
		04UD4JZ
		05U93JZ
		02UR3JZ
		04US4JZ
		04UR3JZ
		03U30JZ
		05UR3JZ
		05UT3JZ
		04UD3JZ
		03U14JZ
		05U90JZ
		04U84JZ
		04U60JZ
		04UV4JZ
		06UY4JZ
		04U73JZ
		03UJ0JZ
		04UC3JZ
		04UB4JZ
		03UD4JZ
		03UL3JZ

© 2023 Kim KM et al. *JAMA Network Open.*

		04UE4JZ
		05UV3JZ
		04UM3JZ
		04UJ0JZ
		03U83JZ
		04U90JZ
		05U13JZ
		04UQ4JZ
		06UC4JZ
		03UV3JZ
		05U33JZ
		05UR4JZ
		04U54JZ
		03UQ4JZ
		03U13JZ
		03U63JZ
		03U94JZ
		06UB0JZ
		06UB4JZ
		02UW0JZ
		03UT4JZ
		06U03JZ
		05UN4JZ
		06UM4JZ
		06US3JZ
		04UC4JZ
		05UL0JZ
		04UW3JZ
		03U53JZ
		03UF3JZ
		04U33JZ
		04UE3JZ
		05UY4JZ
		06U00JZ
		04U30JZ
		05UD0JZ
		04U23JZ
		05U73JZ
		03UU0JZ
		06UG4JZ
		04UB0JZ
		03UP3JZ
		02UT3JZ
		05U40JZ
		04UK0JZ
		04US3JZ
		03U04JZ
		03US3JZ
		06UH3JZ
		02US3JZ
		05UF0JZ
		03UN3JZ
		05UV0JZ
		03UQ0JZ

© 2023 Kim KM et al. *JAMA Network Open.*

		05UL3JZ
		05UC3JZ
		03UK4JZ
		06UJ0JZ
		04UP3JZ
		04UT4JZ
		02UP4JZ
		03U73JZ
		03UK0JZ
		05U30JZ
		04U80JZ
		04U93JZ
		06UC0JZ
		03UV4JZ
		05UH3JZ
		04UN4JZ
		04UL3JZ
		05U50JZ
		05UP0JZ
		04UC0JZ
		03UV0JZ
		05U03JZ
		06US0JZ
		06U64JZ
		06UF0JZ
		05UB0JZ
		05UC0JZ
		06UP3JZ
		05UP3JZ
		06UP0JZ
		05UA3JZ
		06U33JZ
		06UR0JZ
		02UR4JZ
		06UY3JZ
		03UK3JZ
		03U34JZ
		04UQ3JZ
		05UM0JZ
		02UQ0JZ
		05UT0JZ
		04UV3JZ
		06U70JZ
		03UA3JZ
		06U53JZ
		05U64JZ
		04UA3JZ
		04U04JZ
		03US0JZ
		05U83JZ
		03UG3JZ
		03UD0JZ
		03U10JZ
		03UM3JZ

				06U63JZ<br>04U44JZ<br>05UB4JZ<br>03UA4JZ<br>05UG3JZ<br>02UR0JZ<br>04UV0JZ<br>05US3JZ<br>03U50JZ<br>04UY0JZ<br>05UG4JZ
Cardiac	Ligation of Vessel	occlude aorta nec	38.84	04L03ZZ<br>04L04ZZ<br>04L03DZ<br>04L04CZ<br>04L00ZZ<br>04L00DZ<br>04L04DZ<br>04L03CZ<br>04L00CZ
Thoracic		occlude thoracic ves nec	38.85	05L63CZ<br>05L60DZ<br>03L00DZ<br>03L14DZ<br>03L30DZ<br>03L43DZ<br>05L33ZZ<br>03L23DZ<br>05L30DZ<br>03L00CZ<br>02VR3CZ<br>05L50DZ<br>03L34ZZ<br>02LT4ZZ<br>03L33DZ<br>02LT0CZ<br>03L33ZZ<br>02LS0DZ<br>05L54DZ<br>03L43CZ<br>05L30ZZ<br>05L10ZZ<br>02LR0ZT<br>03L03ZZ<br>05L63ZZ<br>03L23CZ<br>03L34CZ<br>05L14DZ<br>05L44DZ<br>03L13CZ<br>03L44CZ<br>02LT4CZ<br>05L34CZ<br>05L00DZ

© 2023 Kim KM et al. *JAMA Network Open.*

		03L14CZ
		05L53CZ
		05L33DZ
		05L03CZ
		02LR4CT
		03L20DZ
		05L34DZ
		03L33CZ
		03L44DZ
		05L53DZ
		05L13ZZ
		02LT0ZZ
		03L24ZZ
		03L34DZ
		05L04ZZ
		03L23ZZ
		05L50CZ
		02LS0CZ
		02LT3DZ
		05L00CZ
		02LS3DZ
		02LR0CT
		05L40CZ
		03L20ZZ
		03L00ZZ
		05L43DZ
		03L43ZZ
		02LS0ZZ
		02LT3ZZ
		02LR4ZT
		03L24CZ
		05L60ZZ
		03L04ZZ
		03L30CZ
		05L43CZ
		05L44ZZ
		02LS3CZ
		02LS3ZZ
		02VQ0CZ
		05L54ZZ
		05L13DZ
		02LR3CT
		02LS4CZ
		03L10ZZ
		03L04DZ
		03L13DZ
		03L20CZ
		05L14ZZ
		05L13CZ
		03L44ZZ
		03L40DZ
		02LT0DZ
		03L10CZ
		05L03ZZ

© 2023 Kim KM et al. *JAMA Network Open.*

				05L64CZ<br>03L03CZ<br>05L30CZ<br>02LS4ZZ<br>05L04CZ<br>05L44CZ<br>03L40CZ<br>05L34ZZ<br>05L40DZ<br>05L33CZ<br>05L00ZZ<br>02VR0CZ<br>05L40ZZ<br>05L64ZZ<br>02LR3DT<br>02LR0DT<br>03L14ZZ<br>02LS4DZ<br>03L30ZZ<br>02VQ3CZ<br>05L43ZZ<br>03L04CZ<br>02VR4CZ<br>05L53ZZ<br>02VQ4CZ<br>05L14CZ<br>03L10DZ<br>02LR4DT<br>05L54CZ<br>05L10CZ<br>02LT4DZ<br>05L64DZ<br>05L50ZZ<br>03L40ZZ<br>03L24DZ<br>05L04DZ<br>05L10DZ<br>03L13ZZ<br>02LR3ZT<br>02LT3CZ<br>03L03DZ<br>05L60CZ<br>05L03DZ<br>05L63DZ
General	Procedures on spleen	total splenectomy	41.5	07TP0ZZ
General	Gastrectomy	proximal gastrectomy	43.5	0DB40ZZ
		distal gastrectomy	43.5	0DB43ZZ<br>0DB44ZZ<br>0DB47ZZ<br>0DT40ZZ<br>0DT44ZZ<br>0DT47ZZ<br>0DT48ZZ
		part gastrec w jej anast	43.7	0D160ZA

© 2023 Kim KM et al. *JAMA Network Open.*

				0D164ZA
				0D168ZA
				0DB60ZZ
				0DB63ZZ
				0DB64ZZ
				0DB67ZZ
				0DB68ZZ
		partial gastrectomy nec	43.89	0DB67ZZ
			43.89	0DB63ZZ
				0DB60ZZ
		total gastrectomy nec	43.99	0DT60ZZ
				0DT67ZZ
				0DT64ZZ
				0DT68ZZ
General	Small bowel procedure	small bowel incision nec	45.02	0D9B8ZZ
				0D984ZZ
				0DCB4ZZ
				0D983ZZ
				0D9B7ZZ
				0D9830Z
				0D9A7ZZ
				0D9B4ZZ
				0D9A4ZZ
				0D9B00Z
				0DC84ZZ
				0D9800Z
				0D9B40Z
				0D9A30Z
				0D9A8ZZ
				0D9A3ZZ
				0D9A00Z
				0D9B0ZZ
				0D987ZZ
				0D9A0ZZ
				0DC83ZZ
				0D9B3ZZ
				0D9B30Z
				0D988ZZ
				0D980ZZ
				0DCA3ZZ
				0DCA0ZZ
				0DC80ZZ
				0DCA4ZZ
				0DCB0ZZ
				0D9840Z
				0D9A40Z
				0DCB3ZZ
		oth excise duodenum les	45.31	0DB97ZZ
				0DB90ZZ
				0DB93ZZ
		local excis sm bowel nec	45.33	0DB80ZZ
				0DB83ZZ
				0DB88ZZ
				0DB87ZZ

© 2023 Kim KM et al. *JAMA Network Open.*

				0DB84ZZ
		mult seg sm bowel excis	45.61	0DB87ZZ
				0DB84ZZ
				0DB80ZZ
				0DB88ZZ
				0DB83ZZ
		part sm bowel resect nec	45.62	0DT98ZZ
				0DT90ZZ
				0DTB8ZZ
				0DTB0ZZ
				0DT94ZZ
				0DTA4ZZ
				0DTB7ZZ
				0DTA0ZZ
				0DTA7ZZ
				0DTB4ZZ
				0DTA8ZZ
				0DT97ZZ
		sm-to-sm bowel anastom	45.91	0D1A8ZA
				0D1B8ZB
				0D1A0ZB
				0D1A8ZB
				0D1B0ZB
				0D1B4ZB
				0D190ZB
				0D194Z9
				0D198ZA
				0D198ZB
				0D194ZB
				0D190Z9
				0D1A4ZA
				0D1B8ZH
				0D1A4ZB
				0D194ZA
				0D190ZA
				0D1A8ZH
				0D1A0ZA
				0D198Z9
		sm bowel-rect stump anas	45.92	0D1A0ZP
				0D1H8ZP
				0D1B0ZP
				0D1B8ZP
				0D1B4ZP
				0D1A4ZP
				0D1A8ZP
		small-to-large bowel nec	45.93	0D1A8ZN
				0D1B4ZK
				0D1A0ZH
				0D1A0ZM
				0D1A8ZM
				0D1B8ZN
				0D1A4ZL
				0D1B8ZL
				0D1A4ZK

© 2023 Kim KM et al. *JAMA Network Open.*

				0D1B4ZL
				0D1B0ZK
				0D194ZL
				0D1B8ZM
				0D190ZL
				0D1B4ZM
				0D1B4ZH
				0D1B0ZL
				0D1A8ZK
				0D1B0ZN
				0D1B0ZH
				0D1B4ZN
				0D1A8ZL
				0D1A4ZN
				0D1A4ZM
				0D1B8ZK
				0D198ZL
				0D1A0ZK
				0D1A0ZN
				0D1A0ZL
				0D1B0ZM
				0D1A4ZH
		anal anastomosis	45.95	0D1B4ZQ
				0D1B8ZQ
				0D1B0ZQ
		duodenal lacerat suture	46.71	0DQ97ZZ
				0DQ93ZZ
				0DQ98ZZ
				0DQ94ZZ
				0DQ90ZZ
		duodenal fistula closure	46.72	0DQ94ZZ
				0DQ90ZZ
				0DQ93ZZ
				0DQ97ZZ
				0DQ98ZZ
		close sm bowel fist nec	46.74	0DQE4ZZ
				0DQ83ZZ
				0DQA8ZZ
				0DQE3ZZ
				0DQ87ZZ
				0DQP7ZZ
				0DQN3ZZ
				0DQB3ZZ
				0DQ83ZZ
				0DQN7ZZ
				0DQB0ZZ
				0HQ7XZZ
				0DQB4ZZ
				0DQ88ZZ
				0DQA7ZZ
				0DQN8ZZ
				0HQ6XZZ
				0DQ88ZZ
				0DQE7ZZ

© 2023 Kim KM et al. *JAMA Network Open.*

				0DQN4ZZ
				0DQA3ZZ
				0DQP0ZZ
				0DQN0ZZ
				0DQP3ZZ
				0DQB0ZZ
				0DQB3ZZ
				0DQ87ZZ
				0DQB7ZZ
				0DQE8ZZ
				0DQ84ZZ
				0DQ80ZZ
				0DQP8ZZ
				0DQ80ZZ
				0DQE0ZZ
				0DQ84ZZ
				0DQP4ZZ
				0DQB7ZZ
				0DQA4ZZ
				0DQA0ZZ
				0DQB4ZZ
				0DQB8ZZ
				0DQB8ZZ
		repair of intestine nec	46.79	0DQE0ZZ
				0DQE8ZZ
				0DQE4ZZ
				0DQE7ZZ
				0DQ93ZZ
				0DQE3ZZ
				0DQ90ZZ
				0DQ94ZZ
				0DQ98ZZ
				0DQ97ZZ
General	Colorectal procedure	tot intra-abd colectomy#	45.80	
		abd-perineal rect resect#	48.50	0DTP4ZZ
				0DTP0ZZ
				0D1N0Z4
				0DTP7ZZ
				0DTP8ZZ
		opn mul seg lg intes nec	45.71	0DBE7ZZ
				0DBE8ZZ
				0DBE0ZZ
				0DBE3ZZ
		open cecectomy nec	45.72	0DTH7ZZ
				0DTH8ZZ
				0DTH0ZZ
		opn rt hemicolectomy nec	45.73	0DTF8ZZ
				0DTF0ZZ
				0DTK0ZZ
				0DTF7ZZ
		opn transv colon res nec	45.74	0DTL8ZZ
				0DTL0ZZ
				0DTL7ZZ
		opn lft hemicolectmy nec	45.75	0DTG8ZZ

© 2023 Kim KM et al. *JAMA Network Open.*

				0DTG7ZZ
				0DTG0ZZ
		open sigmoidectomy nec	45.76	0DTN8ZZ
				0DTN7ZZ
				0DTN0ZZ
		prt lg intes exc nec/nos	45.79	0DBE0ZZ
				0DBE3ZZ
				0DBE7ZZ
				0DBE8ZZ
		lg-to-lg bowel anastom	45.94	0D1K4ZN
				0D1H8ZL
				0D1M8ZM
				0D1N4ZN
				0D1K8ZM
				0D1M8ZN
				0D1L0ZM
				0D1H8ZM
				0D1K0ZK
				0D1M4ZN
				0D1L4ZM
				0D1M4ZM
				0D1K0ZP
				0D1K8ZL
				0D1L8ZN
				0D1K4ZL
				0D1M4ZP
				0D1M0ZM
				0D1K0ZL
				0D1L8ZM
				0D1H4ZN
				0D1H0ZH
				0D1H4ZP
				0D1H0ZM
				0D1K4ZP
				0D1H8ZN
				0D1L0ZL
				0D1H0ZP
				0D1H8ZH
				0D1L4ZN
				0D1N0ZN
				0D1K0ZM
				0D1H8ZK
				0D1L8ZL
				0D1N0ZP
				0D1H0ZL
				0D1H4ZL
				0D1M8ZP
				0D1N4ZP
				0D1L0ZN
				0D1K4ZM
				0D1H0ZK
				0D1L4ZL
				0D1H4ZH
				0D1K0ZN

© 2023 Kim KM et al. *JAMA Network Open.*

				0D1H4ZK
				0D1L4ZP
				0D1K8ZN
				0D1K4ZK
				0D1K8ZK
				0D1L8ZP
				0D1L0ZP
				0D1H4ZM
				0D1M0ZN
				0D1N8ZN
				0D1H0ZN
				0D1K8ZP
				0D1M0ZP
				0D1N8ZP
		suture lg bowel lacerat	46.75	0DQK3ZZ
				0DQE7ZZ
				0DQH8ZZ
				0DQN4ZZ
				0DQK8ZZ
				0DQN0ZZ
				0DQK4ZZ
				0DQE4ZZ
				0DQN3ZZ
				0DQE8ZZ
				0DQK7ZZ
				0DQE3ZZ
				0DQK0ZZ
				0DQH0ZZ
				0DQN8ZZ
				0DQH4ZZ
				0DQN7ZZ
				0DQE0ZZ
				0DQH7ZZ
				0DQH3ZZ
		revise lg bowel anastom	46.94	0DQE3ZZ
				0DQE4ZZ
				0DQE0ZZ
				0DQE7ZZ
				0DQE8ZZ
		ant rect resect w colost	48.62	0DTP0ZZ
				0DTP4ZZ
				0D1N0Z4
				0D1N4Z4
		anterior rect resect nec	48.63	0DTP4ZZ
				0DTP0ZZ
General	Hernia repair (other than inguinal and femoral)	abd repair-diaphr hernia#	53.70	
		thor rep-diaph hern nos	53.80	0BQS0ZZ
				0BQR3ZZ
				0BQR4ZZ
				0BQR0ZZ
				0BQS4ZZ
				0BQS3ZZ

© 2023 Kim KM et al. *JAMA Network Open.*

Thoracic	Therapeutic esophageal procedures	esophageal incision nec	42.09	0D954ZZ<br>0D9570Z<br>0D958ZZ<br>0D9540Z<br>0D953ZZ<br>0D950ZZ<br>0DC53ZZ<br>0D9530Z<br>0DC54ZZ<br>0DC50ZZ<br>0D957ZZ<br>0D9580Z
		cervical esophagostomy	42.11	0D114J4<br>0D11474<br>0D114Z4<br>0D113J4<br>0D11074<br>0D114K4<br>0D110J4<br>0D110Z4<br>0D110K4
		esophagectomy nos	42.40	0DB53ZZ<br>0DB50ZZ<br>0DB57ZZ
		partial esophagectomy	42.41	0DB57ZZ<br>0DB50ZZ<br>0DB53ZZ
		total esophagectomy	42.42	0DT50ZZ<br>0DT57ZZ<br>0DT54ZZ<br>0DT58ZZ
		thorac esophagogastrost	42.52	0D158Z6<br>0D158K6<br>0D158J6<br>0D154K6<br>0D15076<br>0D154J6<br>0DX60Z5<br>0D150Z6<br>0DX64Z5<br>0D150K6<br>0D150J6<br>0D154Z6<br>0D15876<br>0D15476
		suture esophageal lacer	42.82	0DQ54ZZ<br>0DQ57ZZ<br>0DQ58ZZ<br>0DQ53ZZ<br>0DQ50ZZ
		esophag stricture repair	42.85	0D744ZZ<br>0D744DZ<br>0D754ZZ

© 2023 Kim KM et al. *JAMA Network Open.*

				0D740DZ
				0D753DZ
				0D754DZ
				0D750ZZ
				0D743ZZ
				0D740ZZ
				0D753ZZ
				0D750DZ
				0D743DZ
		esophageal repair nec	42.89	0DQ53ZZ
				0DQ54ZZ
				0DQ50ZZ
				0DQ57ZZ
				0DQ58ZZ
General	Therapeutic gastric procedure	gastroenterostomy nec	44.39	0D160J9
				0D160JA
				0D160KA
				0D160ZA
				0D1687A
				0D168K9
				0D160Z9
				0D1607A
				0D168J9
				0D160K9
				0D168ZA
				0D168KA
				0D16079
				0D16879
				0D168JA
				0D168Z9
		sut gastric ulcer site	44.41	0DQ60ZZ
				0DQ63ZZ
				0DQ64ZZ
				0DQ67ZZ
		suture duoden ulcer site	44.42	0DQ98ZZ
				0DQ97ZZ
				0DQ94ZZ
				0DQ93ZZ
				0DQ90ZZ
		suture gastric lacerat	44.61	0DQ63ZZ
				0DQ60ZZ
				0DQ68ZZ
				0DQ64ZZ
				0DQ67ZZ
		close gastric fistul nec	44.63	0DQE3ZZ
				0DQE4ZZ
				0DQ83ZZ
				0DQA4ZZ
				0DQ68ZZ
				0DQA3ZZ
				0DQ84ZZ
				0DQ63ZZ
				0DQE8ZZ
				0DQA7ZZ

© 2023 Kim KM et al. *JAMA Network Open.*

				0DQ64ZZ
				0DQ60ZZ
				0DQ67ZZ
				0DQA8ZZ
				0DQA0ZZ
				0DQ87ZZ
				0DQE0ZZ
				0DQ80ZZ
				0DQE7ZZ
				0DQ88ZZ
		esophagogastroplasty	44.65	0DQ40ZZ
				0DQ44ZZ
				0DQ43ZZ
				0DQ48ZZ
				0DQ47ZZ
		gastric repair nec	44.69	0DV60DZ
				0DV63DZ
				0DV60ZZ
				0DV64ZZ
				0DV67ZZ
				0DV63CZ
				0DQ63ZZ
				0DV63ZZ
				0DQ60ZZ
				0DV64DZ
				0DV60CZ
				0DQ68ZZ
				0DQ67ZZ
				0DV68ZZ
General	Biliary major	gb-to-intestine anastom	51.32	0F144ZB
				0F140Z3
				0F140ZB
				0F144Z3
				0F144DB
				0F144D3
				0F140DB
				0F140D3
		choledochoenterostomy	51.36	0F194Z3
				0F190D3
				0F194D3
				0F190Z3
		hepatic duct-gi anastom	51.37	0F194ZB
				0F154D3
				0F150Z3
				0F154Z3
				0F160DB
				0F164ZB
				0F164DB
				0F150ZB
				0F184DB
				0F164Z3
				0F184ZB
				0F164D3
				0F150D3

© 2023 Kim KM et al. *JAMA Network Open.*

				0F194DB<br>0F184D3<br>0F154ZB<br>0F160ZB<br>0F150DB<br>0F160Z3<br>0F180DB<br>0F190DB<br>0F190ZB<br>0F180D3<br>0F180ZB<br>0F180Z3<br>0F184Z3<br>0F154DB<br>0F160D3
		bile duct repair nec	51.79	0FQ63ZZ<br>0FQ68ZZ<br>0FQ84ZZ<br>0FQ87ZZ<br>0FQ53ZZ<br>0FQ57ZZ<br>0FQ60ZZ<br>0FQ54ZZ<br>0FQ58ZZ<br>0FQ83ZZ<br>0FQ80ZZ<br>0FQ67ZZ<br>0FQ50ZZ<br>0FQ88ZZ<br>0FQ64ZZ
General	Pancreatic procedure	pancreat cyst marsupiali	52.3	0F9G0ZZ<br>0F9G3ZZ<br>0F9G4ZZ
		int drain pancreat cyst	52.4	0F1D0D3<br>0F1D0DB<br>0F1D0Z3<br>0F1D0ZB<br>0F1D4D3<br>0F1D4DB<br>0F1D4Z3<br>0F1D4ZB
		total pancreatectomy	52.6	0DT90ZZ<br>0DT94ZZ<br>0DT97ZZ<br>0DT98ZZ<br>0FTG0ZZ<br>0FTG4ZZ
		rad pancreaticoduodenect	52.70	0D1607A<br>0D160JA<br>0D160KA<br>0D160ZA<br>0DT90ZZ<br>0DT90ZZ<br>0F190Z3

© 2023 Kim KM et al. *JAMA Network Open.*

				0F1G0ZC
				0FTG0ZZ
				0FTG0ZZ
		pancreatotomy nec	52.09	0FFD4ZZ
				0FCG0ZZ
				0FCD7ZZ
				0FFD3ZZ
				0FFD0ZZ
				0F9G3ZZ
				0F9D4ZZ
				0F9D3ZZ
				0F9D8ZZ
				0F9D0ZZ
				0FCG4ZZ
				0FCG3ZZ
				0FFD8ZZ
				0F9G4ZZ
				0FFD7ZZ
				0F9G0ZZ
				0F9D7ZZ
				0FCD0ZZ
		other destru pancrea les	52.22	0FTD0ZZ
				0F5D7ZZ
				0FBD0ZZ
				0FTD7ZZ
				0F5D0ZZ
				0F5G3ZZ
				0FBG0ZZ
				0F5G0ZZ
				0F5D3ZZ
				0FBD3ZZ
				0FBD7ZZ
				0FBG3ZZ
		distal pancreatectomy	52.52	0FBG4ZZ
				0FBG0ZZ
				0FBG3ZZ
		rad subtot pancreatectom	52.53	0FBG4ZZ
				0FBG0ZZ
				0FBG3ZZ
		partial pancreatect nec	52.59	0FBG4ZZ
				0FBG3ZZ
				0FBG0ZZ
		pancreatic anastomosis	52.96	0F1G0D3
				0F1D0D3
				0F1G4ZB
				0F1G4D3
				0F1G4DB
				0F1D4DB
				0F1G0ZB
				0F1D4ZB
				0F1D4D3
				0F1D0Z3
				0F1D0ZB
				0F1D0DB

© 2023 Kim KM et al. *JAMA Network Open.*

				0F1G4Z3<br>0F1D4Z3<br>0F1G0DB<br>0F1G0Z3
General	Liver major	hepatic lobectomy	50.3	0FT10ZZ<br>0FT14ZZ<br>0FT20ZZ<br>0FT24ZZ
		partial hepatectomy	50.22	0FB00ZZ<br>0FB04ZZ<br>0FB03ZZ
Urology	Nephrectomy	nephroureterectomy	55.51	0TT00ZZ<br>0TT04ZZ<br>0TT14ZZ<br>0TT10ZZ<br>0TT68ZZ<br>0TT67ZZ<br>0TT70ZZ<br>0TT64ZZ<br>0TT78ZZ<br>0TT74ZZ<br>0TT04ZZ<br>0TT14ZZ<br>0TT60ZZ<br>0TT00ZZ<br>0TT77ZZ<br>0TT10ZZ
		bilateral nephrectomy	55.54	0TT24ZZ<br>0TT20ZZ
Urology	Major therapeutic procedures of urinary tract	form cutan ileoureterost	56.51	0T164ZC<br>0T1707C<br>0T170JC<br>0T1747C<br>0T184JC<br>0DBB0ZZ<br>0T184KC<br>0T180KC<br>0T174KC<br>0T164KC<br>0DBB4ZZ<br>0T160KC<br>0T174ZC<br>0T174JC<br>0T1847C<br>0DBB7ZZ<br>0T164JC<br>0T184ZC<br>0T160KC<br>0T1607C<br>0T170ZC<br>0T1647C<br>0T184KC<br>0T160JC

				0T160ZC
				0T1807C
				0T1847C
				0DBB8ZZ
				0T170KC
				0T174KC
				0T164KC
				0T1807C
				0T180ZC
				0DBB3ZZ
				0T1747C
				0T180KC
				0T1607C
				0T170KC
				0T1707C
				0T1647C
				0T180JC
		radical cystectomy	57.71	0VT30ZZ
				0TTB4ZZ
				0TTD8ZZ
				0TTB0ZZ
				0TTD7ZZ
				0VT07ZZ
				0VT34ZZ
				0TTB8ZZ
				0TTB7ZZ
				0VT08ZZ
				0VT04ZZ
				0VT00ZZ
				0TTD0ZZ
				0TTB4ZZ
				0TTB7ZZ
				0TTB8ZZ
				0TTD4ZZ
				0TTB0ZZ
		suture bladder lacerat	57.81	0TQB8ZZ
				0TQB3ZZ
				0TQB0ZZ
				0TQB4ZZ
				0TQB7ZZ
		enterovesico fist repair	57.83	0DQP3ZZ
				0DQN0ZZ
				0TQB7ZZ
				0DQP7ZZ
				0DQP0ZZ
				0DQE0ZZ
				0TQB8ZZ
				0UQG8ZZ
				0DQP8ZZ
				0TQD7ZZ
				0DQB3ZZ
				0DQP4ZZ
				0DQB4ZZ
				0TQB4ZZ

		0UQG4ZZ
		0DQN0ZZ
		0DQP8ZZ
		0UQG7ZZ
		0DQB0ZZ
		0DQP4ZZ
		0TQB7ZZ
		0TQB8ZZ
		0DQP7ZZ
		0DQN8ZZ
		0DQN7ZZ
		0DQN4ZZ
		0TQB0ZZ
		0DQ87ZZ
		0TQB4ZZ
		0TQB8ZZ
		0DQE8ZZ
		0TQB8ZZ
		0DQN3ZZ
		0DQN7ZZ
		0DQE3ZZ
		0DQB7ZZ
		0TQB3ZZ
		0UQG3ZZ
		0DQN4ZZ
		0TQB7ZZ
		0DQP3ZZ
		0TQD3ZZ
		0DQN3ZZ
		0DQP0ZZ
		0DQE4ZZ
		0TQB4ZZ
		0DQP3ZZ
		0DQB8ZZ
		0UQG0ZZ
		0DQN8ZZ
		0TQB3ZZ
		0TQB3ZZ
		0DQP8ZZ
		0TQD8ZZ
		0TQD0ZZ
		0TQB0ZZ
		0TQB7ZZ
		0DQE7ZZ
		0DQP0ZZ
		0TQB0ZZ
		0DQ88ZZ
		0TQD4ZZ
		0DQP4ZZ
		0DQP7ZZ
		0TQB4ZZ
		0TQB0ZZ
		0DQ80ZZ
		0DQ84ZZ

© 2023 Kim KM et al. *JAMA Network Open.*

				0TQB3ZZ<br>0DQ83ZZ
		perivesical incision nec	59.19	0WJJ0ZZ<br>0TJB0ZZ<br>0WJJ3ZZ<br>0TJB4ZZ<br>0WJJ4ZZ
Gynecology	Major theraptueic procedures; female organs	pelvic evisceration	68.8	0DTN0ZZ<br>0DTP0ZZ<br>0TTB0ZZ<br>0TTB0ZZ<br>0TTD0ZZ<br>0TTD0ZZ<br>0UT20ZZ<br>0UT20ZZ<br>0UT70ZZ<br>0UT70ZZ<br>0UT90ZZ<br>0UT90ZZ<br>0UTC0ZZ<br>0UTC0ZZ<br>0UTG0ZZ<br>0UTG0ZZ
Orthopedics	Amputation of lower extremity	disarticulation of hip	84.18	0Y680ZZ<br>S53499A<br>0Y670ZZ
		hindquarter amputation	84.19	0Y620ZZ<br>S56919A<br>0Y640ZZ<br>0Y630ZZ<br>S53409A
Transplant	Ot orgn tran	heart transplantation#	37.50	
		heart transplantation	37.51	02YA0Z2<br>02YA0Z0<br>02YA0Z1
		liver transplant nec	50.59	0FY00Z0<br>0FY00Z1<br>0FY00Z2

[b] ICD-9-CM codes for the list of high-risk operations were developed by Schwarze et al (Schwarze ML, Barnato AE, Rathouz PJ, et al. Development of a list of high-risk operations for patients 65 years and older. *JAMA Surg*. 2015;150(4):325). ICD-9-CM codes were converted to ICD-10-PCS using the equivalence mapping developed by the Centers for Medicare & Medicaid Services. The conversion files used were developed by National Bureau of Economic Research and accessed from https://www.nber.org/research/data/icd-9-cm-and-icd-10-cm-and-icd-10-pcs-crosswalk-or-general-equivalence-mappings on August 21, 2021. Conversion and development of this document were done by the authors.

© 2023 Kim KM et al. *JAMA Network Open.*
