# Supplementary material for: Patient Characteristics Associated With Occurrence of Preoperative Goals-of-Care Conversations
Source: JAMA Netw Open. 2023 Feb 9;6(2):e2255407. doi: 10.1001/jamanetworkopen.2022.55407 (PMC9912129; doi:10.1001/jamanetworkopen.2022.55407)
Supplement: Supplement 2. — Data Sharing Statement [file jamanetwopen-e2255407-s002.pdf]

Data Sharing Statement

Kim. Patient Characteristics Associated With Occurrence of Preoperative Goals-of-Care Conversations. *JAMA Netw Open*. Published February 09, 2023. doi:10.1001/jamanetworkopen.2022.55407

Data
Data available: No
